# How Healthy Are Non-Traditional Dietary Proteins? The Effect of Diverse Protein Foods on Biomarkers of Human Health

**DOI:** 10.3390/foods11040528

**Published:** 2022-02-11

**Authors:** Caroline Bull, Damien Belobrajdic, Sara Hamzelou, Darren Jones, Wayne Leifert, Rocío Ponce-Reyes, Netsanet Shiferaw Terefe, Gemma Williams, Michelle Colgrave

**Affiliations:** 1 CSIRO Health & Biosecurity, Adelaide, SA 5000, Australia; damien.belobrajdic@csiro.au (D.B.); sara.hamzelou@csiro.au (S.H.); darren.jones@csiro.au (D.J.); wayne.leifert@csiro.au (W.L.); gemma.williams@csiro.au (G.W.); 2 CSIRO Land and Water, Dutton Park, QLD 4102, Australia; rocio.poncereyes@csiro.au; 3 CSIRO Agriculture and Food, Werribee, VIC 3030, Australia; netsanet.shiferawterefe@csiro.au; 4 CSIRO Agriculture and Food, St Lucia, QLD 4067, Australia; michelle.colgrave@csiro.au

**Keywords:** dietary protein, complementary protein, algae, cereal, grain, fresh fruit, vegetable, insect, snail, mycoprotein, nuts, oil seeds, legume

## Abstract

Future food security for healthy populations requires the development of safe, sustainably-produced protein foods to complement traditional dietary protein sources. To meet this need, a broad range of non-traditional protein foods are under active investigation. The aim of this review was to evaluate their potential effects on human health and to identify knowledge gaps, potential risks, and research opportunities. Non-traditional protein sources included are algae, cereals/grains, fresh fruit and vegetables, insects, mycoprotein, nuts, oil seeds, and legumes. Human, animal, and in vitro data suggest that non-traditional protein foods have compelling beneficial effects on human health, complementing traditional proteins (meat/poultry, soy, eggs, dairy). Improvements in cardiovascular health, lipid metabolism, muscle synthesis, and glycaemic control were the most frequently reported improvements in health-related endpoints. The mechanisms of benefit may arise from their diverse range of minerals, macro- and micronutrients, dietary fibre, and bioactive factors. Many were also reported to have anti-inflammatory, antihypertensive, and antioxidant activity. Across all protein sources examined, there is a strong need for quality human data from randomized controlled intervention studies. Opportunity lies in further understanding the potential effects of non-traditional proteins on the gut microbiome, immunity, inflammatory conditions, DNA damage, cognition, and cellular ageing. Safety, sustainability, and evidence-based health research will be vital to the development of high-quality complementary protein foods that enhance human health at all life stages.

## 1. Introduction

Future food security and population health rely on the development of safe, reliable, and sustainably-produced protein foods to complement more traditional dietary protein sources such as meat, poultry, eggs, and dairy. Population growth, in parallel with a strong consumer demand for non-meat alternatives, has seen complementary protein foods become one of the fastest growing markets of the last decade. The many factors driving this include animal welfare, climate change, environmental sustainability, ethical and religious beliefs, and health. To meet the demand, a broad range of new food products has been launched into the market at pace, together with an equally diverse range of health claims.

While some protein foods, such as legumes, are supported by many years of compositional, safety, and health research, other sources have little or no data available in humans. The scope for the review was intentionally broad to explore evidence generated across a diverse spectrum of potential protein food sources. Non-traditional dietary sources of protein in the scope included algae, cereals/grains, fresh fruit and vegetables, insects and snails, mycoprotein, nuts, oil seeds, and legumes. The specific aims of this review were to gain an understanding of the current state of research across the spectrum of non-traditional dietary proteins and their potential impact on human health, to identify knowledge gaps and potential risks and to propose future opportunities and research directions.

### Scope of Review

Where available, data from human clinical studies was prioritised. Where human studies were limited (or non existent), data from animal and in vitro studies were included in the analysis. Soy protein was deemed out of scope and excluded from analysis. This was due to the fact that a deep body of literature already exists, including multiple reviews dedicated solely to soy protein and its health-related properties. Allergenicity and toxicity are noted as important areas of research, critical for ensuring the safety of novel foods; however, with a growing body of dedicated, quality studies already in place, these topics were also deemed outside the scope of the present study.

## 2. Materials and Methods

### 2.1. Search Strategy

A pragmatic, iterative approach to searching was taken, with a final search strategy for PubMed decided upon through integrating the prior knowledge of the authorship group, previous review papers, and an explorative search in PubMed.

A preliminary search strategy was further refined and modified for a better balance of precision and recall. A search was carried out by the Librarian (DJ) in PubMed, Web of Science Core Collection, and Google Scholar. After an initial screening of results, it was decided to provide a less restrictive result set which allowed the group to ascertain the state of research in those areas where research has included fewer human subject trials. Search string details are provided in Appendix A.

**Figure 1:**
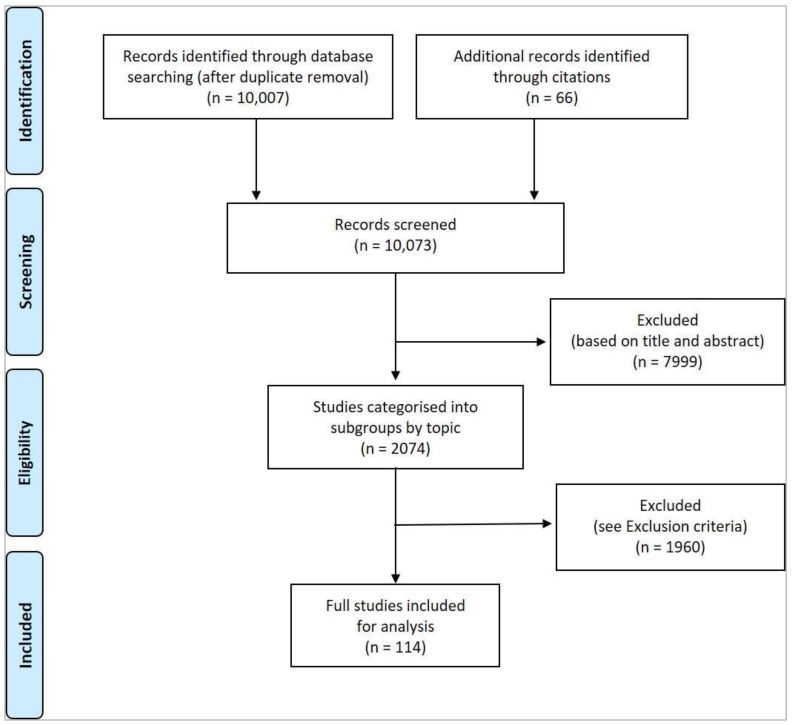
Summary of identification, screening, eligibility and inclusion process [1].

The search process was carried out between 2 September 2021 and 8 September 2021. Results were then deduplicated by the Librarian using the Systematic Review Accelerator Deduplicator tool. Results yielded 10,007 abstracts. A further 66 studies were sourced through the citation listings of review papers. A total of 10,073 studies were screened, with 7999 excluded based on a preliminary review of the title and abstract. The remaining 2074 studies were grouped into topic area for review. Of these, a further 1960 were excluded based on the criteria detailed below. A total of 114 studies were analysed for inclusion in the final synthesis (Figure 1).

### 2.2. Screening and Study Selection

To achieve the aims of the review, and to accommodate the diversity of protein foods, selection criteria were necessarily broad. Where possible, the review focused on high quality human intervention study data. However, at the time of review, some of the more novel protein sources had only animal models or in vitro studies available. Where this was the case, these were considered for analysis.

Inclusion criteria:

- publication date 2000 or later;
- study focus specifically on human health;
- studies examining alternative processing, or ‘raising’, protocols were included only where the outcome was directly focussed on the human health properties of the protein food.

Excluded from analysis were studies:

- relating only to animal health;
- reporting only compositional analysis;
- examining consumer acceptance of non-traditional proteins/foods;
- examining specific non-protein food components (e.g., oils, fibre);
- reporting food frequency data, whole dietary patterns or retrospective dietary analysis;
- reporting the same (or overlapping) data from the same research group;
- conducted in silico;
- review papers, book chapters, or editorials.

## 3. Results

### 3.1. Algal Proteins

Algae have been part of the human diet for thousands of years and provide a wide range of nutrients for health and wellbeing, including vitamins, minerals, dietary fibre, and protein. Algae is consumed whole, as a dried product added to food and drink products, or as a supplement. Search terms included alga, algae, microalgae, macroalgae, and seaweed. After exclusions 14 studies were included for analysis (Supplementary Table S1a,b). There were seven human clinical trials that evaluated the health effects of whole algae, but no clinical trials were conducted on algal protein (Supplementary Table S1a). An additional seven in vitro and animal studies have reported the health effects of whole algae or algal proteins (Supplementary Table S1b).

Clinical studies have shown that whole algae consumption improved a range of metabolic health endpoints [2,3]. Zaharudin et al. showed that the consumption of meals comprising two brown seaweeds (*Laminaria digitata* and *Undaria pinnatifida*) had a lower postprandial glycaemic and insulinemic response and greater satiety compared to an energy-matched meal containing pea protein [2]. A polyphenol-rich brown seaweed, when consumed for eight weeks, provided a modest decrease in DNA damage but only in study participants that were obese [4]. Chronic consumption of *Chlorella vulgaris* (300 mg/day) for eight weeks by people with non-alcoholic fatty liver disease (NAFLD), improved glycemic status as well as C-reactive protein and measures of liver function [3] (Supplementary Table S1a). However, when people consumed 5 g *P. palmata* per day for four weeks, there was no change in blood pressure or serum markers of oxidative stress in either liver or kidney function [5]. In postmenopausal women, *A. exculenta* was shown to alter serum and urine metabolites involving estrogen and phytoestrogen metabolism (REF-Teas et al. 2009), but only a small increase in thyroid stimulating hormone and no difference in other measures of thyroid function [6].

Several in vitro studies have evaluated the cancer-preventive, antioxidant, antitumor, and antihypertensive properties of algae such as *Ulva* sp. and tropical green seaweeds, *Caulerpa racemosa* and *Caulerpa scalpelliformis* [7,8,9,10]. The identification of angiotensin I-converting enzyme (ACE) inhibitory peptides in the protein hydrolysate of the macroalga *Ulva intestinalis*, which are stable during gastrointestinal digestion [9], as well as the ACE inhibitory properties of *Ulva compressa* and *Ulva rigida* [10] make *Ulva* sp. a promising antihypertensive functional food product.

The safety of *Chlorella protothecoides* protein consumption was verified in a 13 week feeding trial in rodents [11] which showed that animals consuming whole algal protein (up to 10% of the diet) had health outcomes, diet intakes, and growth rates that were comparable to a control (casein-based) diet. In another study, spontaneously hypertensive rats that consumed red seaweed *Palmaria palmata* protein hydrolysate (50 mg/kg body weight) showed an acute reduction in blood pressure [12]. Importantly, the renin inhibitory peptide in the seaweed protein hydrolysate remained biologically active following gastrointestinal digestion.

Although there are some promising health effects of consuming algae, it is not clear what components of algae provide these benefits as they could potentially be attributed to dietary fibre, polyphenolics, and other bioactive components such as lipids, proteins, or peptides. There are a small number of preclinical studies reporting the health effects of algae proteins, but clinical studies are currently absent.

The safety of using algae for human and animal food applications also needs to be evaluated carefully given that some algae species can accumulate heavy metals such as cadmium and high concentrations of minerals such as iodine. A range of considerations can be implemented to ensure that the heavy metal and mineral content of algae and algae containing foods remain in the safe range. These include the choice of algae strain and species, the harvesting/growing of algae in a controlled environment, the establishment of quality control regulations to ensure consumer safety, and/or putting limits on the quantity of algae inclusion in food products so that daily intake limits are not exceeded [13,14]. Additionally, processing technologies can be implemented to remove unwanted contaminants in the production of algal extracts [15].

### 3.2. Cereal Proteins

Search terms included wheat, maize, rice, barley, rye, oats, sorghum, quinoa, teff amaranth, and ‘green’ wheat. After screening and exclusions, 18 randomised controlled trials (RCT) were included for analysis (Supplementary Table S2). Most human clinical studies that have evaluated the health effects of cereal proteins used wheat and rice proteins, but rye, corn, oat, and barley proteins have also been studied.

#### 3.2.1. Wheat

In trained cyclists that experienced glycogen depletion protocol, the addition of a mixture of wheat protein hydrolysate and amino acids to a carbohydrate-containing solution (at an intake of 0.8 g carbohydrate per kg per hr) can stimulate glycogen synthesis (Van Loon et al., 2000) [16]. Van Loon et al. (2000) also found that a beverage containing wheat protein hydrolysate, leucine, and phenylalanine resulted in a marked increase in insulin compared with the carbohydrate only drink in fasted cyclists [17]. These studies indicate potential for wheat protein in recovery sports drinks and clinical nutrition.Gorissen et al. (2016) showed that wheat protein hydrolysate increased myofibrillar protein synthesis in healthy older men; however, the rate was not as high as it was when whey protein or casein were ingested [18].

Jenkins et al. (2003) conducted a 1-month RCT in 20 healthy adults to evaluate the effect of high dietary protein intake on calcium balance. The authors concluded that in the presence of high dietary calcium intake (>1.5 g/day), wheat gluten did not have a negative effect on calcium balance, despite increased urinary calcium loss [19].

A 1-month crossover trial (Jenkins et al., 2001) showed that high intakes of wheat protein gluten reduced oxidized LDL, serum triacylglycerol, and uric acid. However, it was not clear whether the effects were due to the higher protein content of the diet or the gluten protein directly [20].

An acute RCT by Stoeger et al. (2019) investigated the effect of wheat protein hydrolysate on measures of satiety in healthy adults. They showed that wheat protein hydrolysate reduced calorie intake from a standardized breakfast. The addition of l-arginine to the wheat protein hydrolysate was more effective in slowing gastric emptying and was suggested to involve increased plasma serotonin levels [21]. Another study by Lee et al. (2016) investigated the effect of adding different levels of wheat gluten to a rye porridge to see if it enhanced measures of satiety, but there was no change in any of the satiety measures evaluated [22].

An acute RCT by Claessens et al. (2009) in eight healthy males compared the postprandial response of plant hydrolysates with maltodextrin. All plant protein hydrolysates induced enhanced plasma insulin and glucagon levels compared to maltodextrin and maintained glucose at low levels. However, the wheat protein hydrolysate showed the lowest increase in plasma glucagon compared to the other protein hydrolysates [23].

#### 3.2.2. Rice

After consuming a single bolus of a rice protein hydrolysate, an RCT by Rein et al. (2019) showed a small but significant suppression of pro-inflammatory cytokines and nitric oxide, whereas IL-6 increased within the 2–12 h following consumption. However, the impact of these changes is not clear given that there was no difference in other cytokines (e.g., IL-10, IL-8, MCP-1) or C-reactive protein (CRP) [24].

A study by Joy et al. (2013) aimed to determine if the post-exercise consumption of rice protein isolate could increase recovery and elicit adequate changes in body composition compared to an equally dosed whey protein isolate if given in large, isocaloric doses. Both whey and rice protein isolate elicited similar improvements in body composition and exercise performance following a resistance exercise program [25]. These findings were supported by Moon et al. (2020) who also showed that rice protein concentrate (24g/day), when consumed for eight weeks along with a resistance training program, led to similar changes in body composition and performance outcomes compared to the consumption of whey protein [26]. Saracino et al. (2020) found that middle-aged men consuming 1.08 ± 0.02 g/kg/day of a rice and pea combination protein did not recover from damaging eccentric exercise after 72 h and that pre-sleep protein ingestion did not aid in muscle recovery when damaging eccentric exercise was performed in the morning compared to whey protein [27].

#### 3.2.3. Barley, Rye and Buckwheat

A study by Jenkins et al. (2010) compared the consumption of bread enriched with barley protein or casein over a 1-month period in 23 hypercholesterolemic men and postmenopausal women. Measures of LDL-cholesterol, C-reactive protein, oxidative stress, and blood pressure were similar for both treatments [28].

Lee et al. (2016) examined the effects of a rye porridge in 21 healthy men and women, compared with a refined wheat bread control. Rye porridge lowered hunger by 20%, desire to eat by 22%, and increased fullness by 29% compared with wheat bread. Plasma glucose after lunch was lower compared with wheat bread. No differences were observed in ad libitum food intake, insulin, or glucagon-like peptides (GLP-1) [22].

Misan et al. (2017) tested buckwheat or corn protein-based porridges. The buckwheat porridge significantly reduced serum levels of total cholesterol, LDL cholesterol, triacylglycerol, and uric acid and significantly increased serum adiponectin levels, HDL cholesterol, and fat-free mass [29].

For pseudo-cereals, animal studies suggest that amaranth hydrolysate may have anti-hypertensive effects [30] and quinoa protein hydrolysate may have antihypertensive effects through blood pressure lowering [31].

Animal studies suggest that buckwheat protein and rice protein isolate may have cholesterol reducing effects through bile acid binding effects [32,33]. Escudero et al. (2006) found hypotriglyceridemic effects and the antioxidant protection of amaranth seed protein compared with casein in rats [34].

### 3.3. Fresh Fruit and Vegetable Proteins

There has been a large commercial growth in animal protein (e.g., whey) fortified functional foods and supplements. In parallel, there has been a keen interest in adopting plant-based protein fortification in-line with vegetarian and vegan diets [35]. In this section, we investigated the effects of dietary proteins from fresh vegetables (and fruit; however, no studies on fruit proteins were obtained). Search terms included fresh fruit and vegetables (potato, fresh peas, beans, sweet potato, cassava, spinach, papaya, tomato, brassica). After exclusions eight studies were included for analysis (Supplementary Table S3). For this section, we found that most human clinical studies examined vegetable protein from potatoes.

Potato protein is viewed as a non-traditional source of plant-derived protein that may provide an alternative to milk and other animal proteins [36]. A byproduct of starch manufacture is potato protein isolate which is relatively cost-efficient to obtain and has other desirable features such as being non-allergenic and gluten and lactose-free [37]. Various studies investigated vegetable protein from potatoes in the form of hydrolysates or extracts and were studied in human clinical trials [38,39,40,41]. In one study, an industrial process was used to isolate native protein fractions from potatoes: termed “high” and “low” molecular weight fractions [39]. These were then tested in an acute, double-blind, cross-over clinical trial in eight healthy adults. Contrary to the effect of casein and whey, the ingestion of 20 g of high or low molecular weight fractions of potato protein isolate did not result in changes to plasma insulin or glucose levels [39].

In a single-blind parallel-group design, 24 women consumed a weight-maintaining baseline diet before being randomized to consume either 25 g of potato protein twice daily or a control diet for two weeks [41]. The study diets consisted of ‘pudding cups’ containing either potato protein (PP) or no protein (Control). Ingestion of potato protein stimulated myofibrillar protein synthesis (from 30–50 mg muscle biopsies) by 0.14 ± 0.09%/day at rest and by 0.32 ± 0.14%/day in the exercising limb, and myofibrillar protein synthesis was significantly elevated by 0.20 ± 0.11%/day in the exercising limb in controls (*p* = 0.008). Another small study showed that potato protein consumption elicited a significantly lower insulinaemic response 30 min after consumption than whey or rice protein. Furthermore, a larger total incremental area under the curve (iAUC) was recorded for whey compared with the potato protein [40].

Amongst the macronutrients, increased consumption of dietary protein is reported to exert the greatest role in appetite control [42,43]. There is limited data on the effect of diverse types or sources of protein on appetite ratings. Dougkas et al. investigated a combination of vegetable protein extracts provided as breakfast supplements in a study with 28 males. The protein sources were semi-skimmed milk (animal protein group) with added milk protein isolate. For the vegetable protein breakfast, the proteins originated from oat drink with the addition of pea and potato protein isolates to increase the essential amino acid concentration and match the quantity and the amino acid profile among the three protein-enriched breakfasts. A further (mixed) breakfast consisted of the oat drink, milk, and pea and potato protein isolates, while the carbohydrate breakfast consisted of rice drink without added proteins. The results showed there were no differences in insulin response or subjective appetite ratings after consumption of the animal protein, vegetable protein, mixture, or the carbohydrate drink (rice drink) [38].

There is limited evidence from early studies identifying the potential satiating capacity of vegetable proteins [37,44,45]. Therefore, studies are needed to elucidate the metabolic benefits of non-traditional sources, such as potato protein, compared to animal (e.g., milk) proteins. Overall, there have been limited studies investigating specific proteins or protein extracts from vegetables (and fruits). There is an opportunity to further explore this area, particularly where food excess/waste or co-product streams can be used to produce protein components for functional foods.

### 3.4. Insect and Snail Proteins

Insects have been part of traditional diets for thousands of years, providing a wide range of dietary nutrients including protein, vitamins, and minerals. Search terms included black soldier fly, cricket, moth, dragonfly, grasshopper, mealworm, silkworm, snail, termite, ant, beetle, and honeybee. After exclusions, 23 studies were included for analysis (Supplementary Table S4). Only four studies were conducted in humans [46,47,48]. Most studies identified were conducted on crickets or mealworms, which are usually eaten whole or ground.

#### 3.4.1. Cricket

Six studies have examined the health effects of cricket (*Gryllodes sigillatus*) consumption, with only one study conducted in people. Following the consumption of 25g of cricket powder per day for two weeks, Stull and colleagues reported that this amount is well tolerated and non-toxic since no significant differences in the gastrointestinal function of the participants relative to the baseline were reported. Additionally, no significant side effects were reported by any of the participants. In terms of phyla-level microbiota composition, operational taxonomic unit (OTU) richness, or Shannon diversity scores no significant changes were observed. However, this amount of cricket powder promoted a significant fold-change of several probiotic taxa: a higher abundance of probiotic bacterium, *Bifidobacterium animalis* (increased 5.7-fold); but *Lactobacillus reuteri* and two other lactic acid-producing bacteria were decreased by 3 to 4-fold [49]. The consumption of cricket powder was also associated with reduced plasma TNF-alpha, a pro-inflammatory cytokine that has been associated with intestinal inflammation and several inflammatory gut conditions [49].

The effect of cricket powder consumption on metabolic health has been examined in rodents. Bergmans et al. (2020) compared the effectiveness of cricket, peanut, and milk proteins in the recovery of malnutrition in a mouse model [50]. Cricket protein showed similar improvements in body weight recovery, but the differences in immune and metabolic markers were inconclusive. No differences were observed between the expression of select inflammatory genes (TLR4, TNFα, IL-1β, IFNγ) in the spleen between the control group and the mice fed cricket or milk diets [50]. Similarly, Oibiokpa et al. (2018) fed a cohort of albino rats isonitrogenous diets containing casein (10% protein) or four different types of insects (cricket, termite, grasshopper or moth) for 28 days. The cricket diet had the highest amino acid score (based on recommended amino acid pattern for preschool aged children) (0.91), protein efficiency ratio (PER, weight gain divided by amount of protein consumed) (1.78), net protein ratio (NPR, weight gain relative to control group) (3.04), and biological value (protein utilization based on absorbed nitrogen) (93.02%). The insect-fed rats’ organ weights (liver, spleen, lung, and heart) were similar to rats fed the control diet. Interestingly, the serum LDL cholesterol concentration was significantly lower in rats fed the cricket diet compared to rats fed with casein and the other insect supplemented diets.

In vitro studies show that cricket powder may have antioxidant, anti-hypertensive, and anti-inflammatory properties. Hall et al. (2020) [51] used a murine macrophage-like cell line to demonstrate that cricket protein hydrolysates contain potent peptides with ACE, α-glucosidase, and α-amylase inhibitory capacity; thus, having potential for lowering inflammation and hypertension. The antioxidant and anti-inflammatory properties of cricket bioactives and the impact of different heat treatment processes was evaluated in a study by Zielinska et al. [52]. They found that baked cricket hydrolysate showed the highest antiradical activity against DPPH (2,2-diphenyl-1-picrylhydrazyl radical) with an IC_50_ value of 10.9 µg/mL [52]. Similarly, Hall et al. (2018) [53] evaluated the effect of enzymatic hydrolysis on the bioactive properties of cricket protein hydrolysates, showing that the bioactivity improved after the simulated gastrointestinal digestion. The authors suggest that the consumption of edible cricket peptides, alone or in the form of functional foods, might contribute to positive effects towards conditions associated with inflammation and hypertension.

#### 3.4.2. Mealworm

Only one human intervention study with mealworms was identified. This was conducted in a small cohort of six males, in which four different supplements (lesser mealworm (*Alphitobius diaperinus*), whey isolates, soy isolate, or water) were tested acutely on four separate days. All three protein isolates were associated with a post-prandial increase in essential amino acids (EAAs), branched chain amino acids, and leucine concentrations in blood over 120 min. AUC analysis showed significantly greater blood amino acid concentrations after whey, than soy or lesser mealworm. Mealworm protein had the highest blood amino acid concentration after 120 min, suggesting the mealworm may to be a ‘slow’ digestible protein source [48].

Several animal studies have tested metabolic biomarkers following supplementation with dietary yellow mealworm protein. Ham et al. [54] compared the effects of low- and high-fat diets comprising protein from either soy or mealworm (de-fatted and freeze-dried fermented yellow mealworm (*Tenebrio molitor*) extract, TMP) in mice. The authors noted that the fermentation process provides higher concentrations of free EAAs. Following a 12-week intervention, TMP-fed animals had lower body weight, weight gain, fat mass, and improved glucose tolerance compared to animals on the soy protein diet. In addition, when TMP was added to the high fat-diet, hepatic steatosis was reduced, and genes associated with lipid and amino acid metabolism and oxidation were down-regulated. Gessner et al. and Seo et al. [55,56] came to similar conclusions from mouse and rat in vivo studies, with results indicating mealworm also reduced triacylglycerol and cholesterol biosynthesis and lowered homocysteine in liver and plasma by up to 30%. The study on 3T3-L1 adipocytes by Seo et al. [56] suggested that these changes may be due, in part, to mealworm larva stimulating the phosphorylation of adenosine monophosphate (AMP)-activated protein kinase and mitogen-activated protein (MAP) kinases. Comparison of mice fed semi-purified diets containing one of six proteins (300 g/kg), including yellow mealworm, showed that protein source has a significant impact on metabolism and metabolic amine profiles (amine metabolites) in serum and urine. Metabolites such as alpha-aminobutyric acid and 1-methylhistidine were shown to be sensitive indicators of too much or too little availability of specific amino acids in the different protein diets [57].

Data from in vitro studies indicate that hydrolysis treatment of yellow mealworm proteins deleteriously impacts its anti-inflammatory activity [58]. Lacroix et al. showed that the enzymatic digestion of lesser mealworm protein with thermolysin was the most effective in releasing active peptides, for both the isolate and the concentrate. Thermolysin-generated hydrolysate contained increased DPP-IV inhibitors, with the potential to modulate incretin levels following meal consumption and reduce blood glucose concentration [59,60]. Zielinska et al. [61] showed that heat treatment had a positive effect on antioxidant and anti-inflammatory properties, and that the peptide fraction from mealworm protein had high iron-chelating activity. Recent data from ex vivo models (pig intestine) and in vitro digestion studies showed that insect and beef were equally effective in reducing the digestive hormone cholecystokinin (CCK) when compared to almond protein. Mealworm protein also reduced the appetite hormone, ghrelin, in both human and pig ex vivo models. However, this observation was not supported by a feeding study in rats that increased their food intake when the diet contained mealworm protein (300 mg/kg) instead of raw almond [62].

#### 3.4.3. Silkworm

Only one study has evaluated the health effect of freeze dried mature silkworm larvae powder (*Bombyx mori*; silkworm). Lee et al. administered silkworm powder to rats, in parallel with oral ethanol gavage, to explore the effect on biomarkers of alcohol-induced fatty liver disease [63,64]. Rats treated with silkworm powder had lower hepatic triglycerides (by 35%), together with a reduced plasma triglycerides and inflammatory markers (tumor necrosis factor-alpha and interleukin-1 beta, cytochrome P450 2E1 generating oxidative stress) compared to those for which ethanol was administered alone. Consistent with these changes, the expression of genes involved with lipogenesis and fatty acid oxidation were upregulated in rats administered with silkworm powder. These findings suggest that silkworm powder may have a protective effect; however, much more research is needed [63].

#### 3.4.4. Termites

There are currently no clinical or animal trials that have explored the health benefits of termite consumption. However, there are reports that termites (*Macrotermes bellicosus*; *M. nigeriensis*) could be included in the human diet given that they are rich in protein (approximately 30%) and minerals (Mg, Ca, K and P) and low in antinutrients (tannins, phytate, saponins and oxalate) [47]. Nursing mothers in Nigeria (*n* = 60) reported high acceptability of common infant foods (maize and sorghum pap, boiled rice, and yam) supplemented with ground *M. bellicosus* (4:1 ration, *w/w*). Compositional analysis showed a significant increase in nutrient and energy content of the study foods, with termite providing 20% of the total (2–6%) protein. In a survey cohort of 700, 94.5% of respondents reported never having a negative effect from consuming *M. bellicosus* [46]. Sensory testing of wheat cakes supplemented with 0–20% milled paste of *M. nigeriensis* indicated that 5% termite is preferred. Protein content ranged from 10–19.5%, and mineral content increased with increasing termite inclusion.

#### 3.4.5. Snails

Protein energy malnutrition is a serious health burden in developing nations, contributing to high rates of childhood morbidity and mortality, most notably in sub-Saharan Africa [65]. Agengo et al. [65] compared supplementation of sorghum wheat buns with 5–25% snail meat (species not stated) or skimmed milk powder, testing nine diets in a weanling rat model, including a rehabilitation diet containing 16% protein. Protein and feed efficiency ratios, as well as apparent and true protein digestibility all significantly increased in snail meat treated animals compared with the skimmed milk group. Protein digestibility corrected amino acid score (PDCAAS) and the digestible indispensable amino acid score (DIAAS) increased from 45% to 78% and 44 to 69%, respectively, in snail meat fortified buns. Snail meat fortification was also effective in promoting growth and rehabilitation of emaciated rats compared to those consuming bun fortified with skimmed milk (control) which suggests that it could be a strategy used in assisting with the recovery of malnourished children [65]. The dietary inclusion of snail meat has been shown to affect metabolic and bone health in two rodent studies. A diabetic mouse model was used to test freshwater snail (*Semisulcospira libertine*) hydrolysate on biomarkers of type-2 diabetes, liver and kidney health [66]. After 12-weeks, snail meat-fed groups (125, 250, and 500 mg/kg) had dose-dependent, significant reduction in all glycaemic control biomarkers and diabetic complications. These included lower blood glucose and insulin concentrations, reduced glucose utilization related to hepatic glucokinase (GK) activity, and an increase in hepatic gluconeogenesis-related phosphoenolpyruvate carboxykinase (PEPCK) and glucose-6-phosphatase (G6pase) activity. The authors note that the inclusion of 125 mg/kg of snail meat in the high-fat diet was similar or more potent than treatment with metformin (250 mg/kg) [66]. However, a study by Radzki et al. [67], conducted in growing rats, showed that snail meat may adversely affect bone health. They showed that when the animals were fed three different species of snail (*Helix pomatia*, *Cornu aspersum maximum*, *C. aspersum aspersum*) at 10% of the diet for 28 days, although bone mineral density was not affected, bone mineral content, area of total skeleton, and tibia resistance to mechanical load was reduced in snail meat fed groups compared with the casein control.

Several invertebrate species, including snails have been shown to elicit anti-hypercholesterolemic activity in an in vitro model. In a study by Huang and colleagues [68], heat-generated protein hydrolysate of *Achatina fulica* foot muscle (121 °C for 60 min) was then further hydrolysed using proteases papain, trypsin, or alcalase. ACE inhibitory activity was tested in the secondary hydrolysis products. Alcalase hydrolysate was effective in disintegrating intact cholesterol micelles and had strong ACE inhibitory activity in vitro [68].

### 3.5. Mycoprotein

After screening and exclusions, nine human clinical studies were included in the final analysis (Supplementary Table S5). Mycoprotein, derived from the fungus *Fusarium venenatum*, was approved in 1984 as a food protein for human consumption and subsequently marketed globally under the brand name Quorn ^TM^. Human clinical studies, using whole foods or freeze-dried isolate, have examined the effects of mycoprotein on health including muscle synthesis and gene expression, biomarkers of glycaemic control, cholesterol, and satiety. Myofibrillar protein synthesis was found to be equivalent for mycoprotein compared with animal protein foods and whey protein in both exercised and rested leg muscle [69]. Mycoprotein stimulated greater post-exercise muscle protein synthesis and was superior in supporting acute tissue remodelling, compared with (lysine-matched) milk protein [70], whereas enrichment of mycoprotein with branched-chain amino acids (BCAA) failed to further enhance muscle protein synthesis [71].

Mycoprotein as whole foods, and in a shake-based dose-response study, showed limited or no difference in glycaemic control measures, compared with meat, fish, or milk protein [72,73,74]. Mycoprotein has a positive effect on satiety, with acute study participants consuming less energy in meals following consumption [75,76]. Mycoprotein does appear, however, to offer significant benefits over traditional protein sources for management of cholesterol levels [72] as well as slower and more sustained postprandial circulating amino acid concentrations [74]. Notably, a 6-week intervention with mycoprotein showed a significant reduction in total and LDL-cholesterol in individuals with high baseline measures [77]. It should be noted that mycoprotein intervention studies have predominantly been acute or short time periods (hours/days) and in relatively small cohorts (10 to 55), suggesting larger studies are warranted.

### 3.6. Nut and Oil Seed Protein

#### 3.6.1. Nuts

Nuts are a valuable source of many nutrients including protein. However, like nearly all plant proteins, they are lacking in some essential amino acids making them an incomplete source of protein. There is interest in using nut proteins as ingredients in functional foods. Search terms included all nut varieties such as almonds, walnuts, hazelnuts, Brazil nuts, pecans, cashews, and pine nuts. While no human studies were identified in the search results, several papers using in vitro methods or in vivo animal models to explore the nutritional properties and health benefits of a variety of nut proteins and their hydrolysates were retrieved. After screening and exclusions, twelve studies were included in the final analysis (Supplementary Table S6a).

Five of the twelve papers relating to nut protein focused on the nutritional content and health effects of walnut protein and its hydrolysates. Wang et al. (2016) displayed the antioxidant and antihypertensive properties of walnut protein and walnut protein hydrolysate in in vitro studies [78]. The other five walnut protein papers were animal studies. Li et al. showed an improvement in renal function in rats [79] while the other studies displayed a boost in immune function in mice [80], improved glycogen reserve, and fatigue recovery in mice [81], memory in mice [82] and anti-photoaging of the skin in rats [83].

Almonds, hazelnuts, cashew nuts, pine nuts, and peanuts have been evaluated in in vitro or animal studies [84]. Anti-hypertensive and anti-inflammatory activity in almonds was studied in in vitro models by Liu et al. (2016) [85] and Udenwigwe et al. (2013) [86]. Ren et al. (2018) found that a peptide from hazelnut proteins exerted anti-inflammatory activity in vitro [87]. The hypoglycaemic activity of Korean pine nut protein was studied in mice by Liv et al. (2019) who concluded that there is potential for pine nut protein to be beneficial as a hypoglycaemic functional food in the treatment of type 2 diabetes mellitus [88]. Cashew nut protein (and their low molecular weight peptides) was investigated in vitro by Malomo et al. (2020) [89]. Their results showed strong antioxidant properties as well as renin-angiotensin system inhibition, which supports further investigation in in vivo trials. While peanuts are classified as legumes, in Australia, they are consumed as nuts and will therefore be covered in this section. Only one study in rats investigated the effect of peanut protein on body composition, lipids, and muscle morphology compared with animal proteins (casein and cod). Jacques et al. (2010) reported that rats fed the diet containing peanut protein had lower muscle mass and body weight compared to the rats fed diets containing casein or cod. It is likely that the poor quality of the peanut protein (low essential and branched chain amino acids) was the cause of the impaired growth of these animals [90].

#### 3.6.2. Oil Seeds

Compared to nuts, seeds are higher in protein and most amino acids [84]. Seeds are also a rich source of healthy fats and many of the nutritional and health effects of nuts and seeds relate to this aspect of their nutritional composition. Search terms included all seed varieties such as canola/rapeseed, hemp seed, chia, sunflower, flaxseed/linseed, pumpkin, and sesame seed. After screening and exclusions, eleven studies were included in the final analysis (Supplementary Table S6b).

The health benefits of canola (rapeseed) protein were investigated in three human clinical trials and an animal study. Bos et al. conducted a human feeding trial on 12 subjects to test the bioavailability and metabolic utilsation of canola protein. They concluded that canola protein is a particularly promising seed protein as it has a high biological value that could be sufficient to meet human requirements for essential and non-essential amino acids [91]. An acute clinical study by Fledderman et al. (2013) showed that when people consumed canola protein isolates and hydrolysates, the postprandial amino acid response was similar to that seen for soy protein. These findings were supported by a study in rats which showed that the canola protein was highly digestible [92,93]. Volk et al. (2020) conducted a randomized controlled cross-over study comparing test meals with additional canola protein isolate or soy protein isolate and found the canola protein had a favourable effect on postprandial insulin and satiety in humans, making canola protein a potentially valuable plant protein for human nutrition [94].

There is also growing interest in the potential antihypertensive and antioxidant effects of hemp seed protein. A feeding study in hypertensive rats showed a blood pressure-lowering effect attributed to specific peptides found in hemp seed meal protein hydrolysates that have antioxidant properties [95]. In support of this, Malomo et al. (2015) tested various hydrolysates of hemp seed protein for in vitro inhibitions of renin and angiotensin-converting enzyme (ACE), two of the enzymes that regulate human blood pressure, and found promising reductions in systolic blood pressure [96]. A clinical trial by Samsikor et al. (2020) is currently exploring the effect of hemp seed protein (and its hydrolysate) on hypertension in humans; however, the results are not available at the time of publication [97].

Sesame seed protein (and its isolate and hydrolysate) is a similarly suitable edible protein compared to casein and soy protein, as determined by Sen et al. (2001) [98]. Two animal studies have evaluated the health effects of sesame seed protein. In one, rats fed a diet containing sesame seed protein had similar levels of plasma protein, liver lipids, and erythrocyte membrane lipid concentrations compared to rats fed diets containing casein or soybean meal [98]. An animal study by Biswas et al. (2010) reported favourable changes in plasma cholesterol (lower total cholesterol and higher HDL-cholesterol) when rats consumed the diet containing sesame protein isolate [99]. A recent study investigated the in vitro antioxidant and antihypertensive potential of a sesame seed protein isolate and its peptide fractions compared to the unhydrolyzed protein. The sesame seed peptides demonstrated superior antioxidative and antihypertensive activity in the form of ACE inhibition [100].

An animal study has investigated the effect of sunflower seed protein on the growth, plasma, and tissue lipid profiles and the plasma protein content, erythrocyte membrane lipid profile, and organ weights of rats. The rats showed little variation in plasma protein content liver and brain lipids but exhibited a superior effect on erythrocyte membrane lipids indicating promising hypolipdemic properties of sunflower seed protein (Sen et al. 2000 [101,102]).

### 3.7. Non-Soy Legume Proteins

As there are extensive clinical studies and systematic reviews reporting on the health effects of soybean proteins, here we have only reviewed studies conducted with non-soy legumes. Search terms included pea, lentil, lupin, legume, bean, and chickpea. After exclusions, 22 RCT studies that reported the health effects of non-soy legumes were included in the analysis (Supplementary Table S7).

#### 3.7.1. Peas

The effect of pea protein and pea protein hydrolysate consumption on satiety, subjective appetite, and postprandial blood glucose was evaluated in seven clinical trials. Diepvens et al. compared the effect of ingesting equivalent amounts (15 g) of pea protein hydrolysate (PPH) with whey protein (WP), whey protein plus pea protein hydrolysate (1:1 mixture, PPH + WP), and milk protein (80% casein, 20% whey, MP) in isocaloric and macronutrient matched test protein shakes. Outcome measures were subjective appetite scores and ad libitum food intake (FI) 3 h after the consumption of these products in a randomized crossover trial involving obese adults (BMI 25–31 kg/m^2^). Overall, no significant effects of the various test meals were observed on FI or hunger scores. However, the PPH shake resulted in a significantly higher satiety and fullness and less desire to eat at various time points up to 3 h compared to MP or PPH+WP shakes [103]. 

In another study, protein hydrolysates from plant sources (pea, gluten, rice, and soy) were compared with protein hydrolysates from animal origins (whey and egg) using a repeated-measures design with Latin square randomization and single-blind trials that examined only eight individuals [23]. The study consisted of seven trials in which six different protein hydrolysates and one control beverage (maltodextrin) were tested. All beverages (approximately 250 mL) offered to the subjects contained 0.2 g protein hydrolysate per kg body weight. Postprandial plasma glucose, glucagon, insulin, and amino acids were determined over 2 h. BCAA concentrations were directly proportional with insulin and glucagon response, and the best predictors; WPI induced the highest insulin response, and soy and gluten the lowest. All protein hydrolysates induced an insulin response significantly greater than control [23].

Abou-Samra et al. (2011) investigated the effect of 6 test beverages incorporating similar amounts (20 g) of different proteins i.e., pea protein, casein, whey protein, egg albumin, or maltodextrin in 200 mL water on subjective satiety and FI 30 min following the ingestion of the beverages in RCT involving 32 healthy young men. FI was significantly lower in the case of the pea protein and casein protein preload, whereas the combined satiety score was significantly higher. A second experiment within the study excluding egg albumin and maltodextrin and ad libtum meal immediately after the beverages showed no significant effect of either of the beverages both on FI and combined satiety score, suggesting that the effects of the protein preload are significant only in cases where there is a time gap between the preload and the meal [104].

In order to determine the specific effects of pea proteins compared to pea fibre, a repeated-measure randomized trial involving 19 and 20 men, respectively, was conducted [105]. The treatment meals were pea protein isolate (P10: 10 g, P20: 20g) and pea hull fibre (F10: 10 g, F20:20 g), all in tomato soup, with tomato soup as control. Participants were given an ad libitum pizza meal 30 min (experiment 1) or 120 min (experiment 2) after the test meals. In the first experiment, P20 resulted in a significantly lower FI compared to all the other treatments suggesting that the impact of protein preload on appetite and subsequent food intake is dependent on amount. In addition, the pre-pizza meal BG was significantly lower in P10 and P20 treatments compared to the control, whereas the post-pizza meal BG was significantly lower in the case of P20 compared to the control and F10. No significant differences in FI and BG were observed between the various treatments in experiment 2, indicating that the positive effect of protein preload on FI and BG is dependent on the time gap between the protein meal and the subsequent meal, supporting the findings of Abou-Samra et al. Moreover, the various treatment meals did not have a significant effect on subjective appetite in both experiments [105]. In a subsequent study, the same investigators [106] compared the impacts of pea hull fibre (7 g), pea protein (10 g), a combination of both, or canned yellow peas (406 g to match the protein and hull fibre in pea) on BG, subjective appetite, and FI 135 min after the test meals. Protein plus fibre and yellow pea consumption led to lower blood glucose compared with the pea fibre group (*p* < 0.05) indicating that both the protein and fibre components contribute synergistically towards the impact of peas consumption on glycaemic control. No differences were observed in FI or appetite measures in agreement with the earlier study [105] by the same group [106].

A study by Tan et al. compared the effects of ingesting 25 g of pea, oat, or rice proteins in a high carbohydrate chocolate beverage with a plain chocolate beverage on BG in RCT involving 30 healthy Chinese men. The pea and oat proteins resulted in a significant increase in postprandial insulin secretions. However, none of the proteins had a significant effect on either BG or appetite [31].

Chauhan et al. (2021) investigated the consumption of AI-designed pea protein hydrolysate (15 g) for 12 weeks on the HBA1c of healthy prediabetic adults (HBA1c: 5.7 to 6.4%). The consumption of pea protein hydrolysate resulted in a significant (but small) reduction (0.12%) in the average HBA1c of the participants, whereas a placebo (containing microcrystalline cellulose, avicel^®^) or rice protein hydrolysate did not have a significant effect [107].

Some studies investigated the effect of pea protein consumption on postprandial blood pressure (BP). Li et al. (2011) studied the effect of consumption of two different doses of pea protein hydrolysate (PPH 0.5 g and 1 g) compared with orange juice three times a day over three weeks in seven hypertensive subjects (systolic BP 120–170 mm Hg). The higher dose of PPH (3 g per day) resulted in a significant reduction of systolic BP of 5- and 6-mm Hg after week two and three, respectively [108]. A randomised cross-over trial involving 48 overweight or obese men with untreated, elevated BP compared the effects of six test meals consisting of 0.6 g per kg body weight of protein or carbohydrate over a period of two weeks. The six test meals contained either pea protein isolate, milk protein isolate, egg white protein isolate, a mixture of pea, milk, egg, and soy protein isolates (20% each), sucrose, or maltodextrin. The consumption of pea protein isolate resulted in a significantly lower diastolic BP compared to the other treatment meals [109].

Weinborn et al. (2015) compared the effect of co-ingestion of heme iron (Fe) with pea protein isolate, lentil protein isolate, or soy protein concentrate on Fe absorption in a study involving 15 female participants. The average heme Fe absorption was 11% when heme Fe was administered alone, and absorption was not significantly affected by co-ingestion with pea and lentil protein isolates, whereas soy protein concentrates significantly decreased absorption to 7.2% (*p* < 0.02) [110].

#### 3.7.2. Lentil

A randomised cross-over trial involving 48 healthy men was conducted to determine the specific component in lentils that contribute to the observed effect of lentils on satiety and postprandial blood glucose [111]. The study included tomato soup as a control and tomato soup incorporating 20 g of lentil protein isolate (75% purity), lentil protein concentrate (55% purity), lentil starch (60% purity), or lentil fibre (55% purity) as test meals. The participants were given the test meals prior to an ad libitum pizza meal 30 min (experiment one) or 120 min (experiment two) after the test meal. In the first experiment, the lentil protein isolate and the lentil protein concentrate meals lowered subjective appetite (*p* < 0.05) and postprandial BG response (*p* < 0.0001). The reduction in BG was even greater for the lentil protein isolate and concentrate in the second experiment when there was a longer time gap between the preload and the pizza meal (120 min rather than 60 min) [111].

#### 3.7.3. Lupin

The effects of lupin proteins on health targets including blood lipid profile, post-prandial BG, immune and oxidative responses were investigated in seven clinical trials. Weisse et al. (2010) compared the effects of a daily snack (35 g/d) containing isoflavone-free lupin protein isolate, or casein (control), on blood lipids in 43 hypercholesteremic subjects over six weeks. Consumption of the lupin snack resulted in a significant reduction of LDL-cholesterol (8.6%) and LDL/HDL ratio. The casein snack resulted in a similar reduction of total cholesterol, mainly due to a significant reduction (10%) in HDL-cholesterol [112].

Bähr et al. (2013) compared the effects of lupin protein (LP) isolate containing small amount of alkaloids and almost free of γ-conglutin (25 g/day) and milk protein isolate (MP) (25 g/day) in a beverage format along with their regular diet on the blood lipid profile of 33 hypercholesterolemic subjects (total cholesterol ≥5.2 mmol/L) for eight weeks. Both interventions resulted in a significant but small reduction in LDL cholesterol (5.9% and 7.3% for LP and MP respectively) after four weeks, although the changes did not last through the eight-week intervention. There was a significant reduction of LDL/HDL in the LP diet after four weeks. For subjects with severe hypercholesterolemia (total cholesterol >6.6 mmol/L), both interventions resulted in a significant reduction of total cholesterol, LDL and LDL/HDL ratio after four weeks intervention, and the change in LDL and LDL/HDL ratio lasted through the eight week intervention period in the case of the LP intervention [113]. In a follow-up study involving 72 hypercholesterolemic subjects, the same research group compared the effect of the two protein isolates when incorporated into complex food products including bread, rolls, scalded sausage, and vegetarian spread on the same end points over 28 days. A third intervention consisting of milk protein isolates and an arginine capsule (MPA) to compensate for the higher level of arginine in lupin was also investigated. The total cholesterol level decreased significantly (*p* < 0.05) by 4.3% and 5.3%, respectively, after the LP and the MPA interventions. Moreover, LDL cholesterol significantly (*p* < 0.05) decreased by 3.6% and 3.8%, respectively, after the LP and MPA interventions, whereas triacylglycerols decreased only after the LP intervention [114]. In contrast to the earlier study, no effect was observed with the MPA intervention, indicating that the effect of lupin on blood lipids could be linked to its higher arginine content [114].

Pavanello et al. (2017) compared the effect of lupin protein concentrate (30 g/day) with lactose-free skimmed milk powder (30 g/day) along with a low-fat normal calorie diet on the blood lipid profile of moderately dyslipidaemic and overweight subjects (*n* = 50). Total cholesterol level significantly decreased to the same degree in both the milk and the lupin diets (6.7% and 7.2% respectively). However, a significant reduction in LDL cholesterol (8% reduction) and non-HDL cholesterol (8% reduction) was observed only in the lupin diet. There was also a significant reduction in proprotein convertase subtilisin/kexin type 9 (PCSK9) (12.7% reduction) in the lupin diet [115].

Cruz-Chamorro et al. (2021) investigated the effects of the consumption of 1 g lupin protein hydrolysate in a beverage format (LPHb) for 28 days on blood lipids and immune and oxidative biomarkers of healthy subjects (*n* = 33). The treatment did not have a significant effect on total cholesterol (TC), LDL cholesterol, HDL cholesterol, and total triglycerides level. On the other hand, a slight (4.2%) but significant (*p* ≤ 0.05) reduction in LDL/HDL ratio was observed due to a significant reduction (*p* ≤ 0.01) in LDL/HDL ratio of the male participants (15% reduction). In addition, a significant reduction (*p* < 0.05) in TC and LDL was observed in males with higher baseline risk factors for CVD (high BMI, high TC, high Caselli risk index I (TC/LDL) and II (LDL/HDL)). The treatment also resulted in a significant reduction of phytohemagglutinin-P (PHA) stimulated production of Th1 pro-inflammatory cytokines IL-2, IFN-γ, and TNF in human peripheral blood mononuclear cells (PBMCs) as well as a significant increase in TAC and ORAC antioxidant capacity of PBMCs of participants [116].

Bertoglio et al. (2011) investigated the effect of various doses (157.5, 315 and 630 mg) of lupin γ-conglutin ingested 30 min prior to a high carbohydrate meal on blood glucose and insulin responses of 15 healthy volunteers over three hours following the meal. A significant reduction of 25% and 21% in blood glucose level calculated as AUC was observed at the intermediate and the highest γ-conglutin doses, respectively. There was no effect on insulin secretion and the authors concluded that γ-conglutin acts as an insulin mimetic based on an earlier cell model studies which showed that the treatment of myocytes with γ-conglutin activates proteins and enzymes in the insulin signaling pathway [117].

#### 3.7.4. Mixed Legumes

Several clinical studies investigated the impact of mixed legume meals on satiety and other health targets. Four studies that satisfied the inclusion criteria are reviewed in this section. Kristensen et al. (2016) compared the impact of high protein fava beans and a peas-based meal (HP-legume) with that of high protein meat-based meal (HP-meat) and low protein fava beans and peas-based meal (LP legume) on subjective appetite sensation and satiety in RCT involving 43 subjects. The two high protein meals were macronutrient and energy matched, whereas the third meal was energy and fat-matched with the two meals. The three meals had a different amount of dietary fibre (6 g, 23 g, and 10 g fibre per 100 g for the HP-meat, HP-legume, and LP-legume, respectively). The HP-legume meal induced a significantly lower (*p* < 0.05) composite appetite score, hunger, prospective food consumption, and a significantly higher (*p* < 0.05) fullness compared to HP-meat and LP-legumes even after compensating for palatability. The HP-legume meal also resulted in a significantly higher satiety compared to the HP-meat meal. In addition, three hours after the HP-legume meal the FI was 12% and 13% lower (*p* < 0.05) compared to the HP-meat and the LP-legume meals, respectively [118]. A subsequent study by the same group compared the impact of four meals viz. fava beans-split peas, pork and veal with pea fibre, egg with pea fibre, and egg-based meals on the same endpoints [119]. The first three meals were macronutrient, energy, and fibre matched, whereas the last meal was macronutrient and energy matched with the three meals. In this case, no significant difference (*p* > 0.05) was observed between the four meals in FI, subjective appetite sensation, composite appetite score, fullness, and prospective food consumption (PFC), indicating that the observation in the earlier investigation could be due to the higher fibre content of the legume meal compared to the meat-based meal when the protein content is kept the same. However, that does not explain the effect of the egg only meal [119].

A study by Dougkas et al. (2018) investigated the effects of four types of test meals viz a rice pudding containing milk protein (AP), mixed vegetable protein (peas, oat, potato; VP), or a mixture of vegetable and milk protein (50:50) (MP) and a carbohydrate-rich control meal, on satiety and FI in RCT involving 28 healthy men. A significantly lower subjective appetite was observed in the case of the MP compared to the control whereas fullness was significantly higher than the control in both MP and VP meals. No significant difference was observed between the test meals in FI after three hours [38].

Hosseinpour-Niazi et al. (2015) investigated the impact of macronutrient, energy, and fibre matched meat–based and legume-based (lentil, peas, beans, chickpeas) intervention diets on blood inflammatory markers (high sensitivity c-reactive protein (Hs-CRP), IL-6, TNF-α) on diabetics in RCT involving 31 participants. The two diets were similar except that legumes replaced two servings of meat over three days a week. Both intervention diets resulted in a significant reduction of Hs-CRP, IL-6, and TNF-α. Nevertheless, a significantly higher reduction was observed in the legume diet (Hs-CRP decreased by 1.3 and 1.7 mg/L respectively, *p* = 0.019, IL-6 decrease by 1.2 and 1.6 pg/L respectively, *p* = 0.018, TNF-α decreased by 1.3 and 1.8 pg/L respectively, *p* = 0.018) perhaps due to the positive effects of the legume proteins and/or other components [120].

## 4. Discussion

The aim of this review was to evaluate the reported preclinical and clinical health effects for a broad range of non-traditional protein foods and identify knowledge gaps, risks, and potential research opportunities. A search strategy was chosen that would capture a broad range of preclinical and clinical research reporting on the health effects of non-traditional proteins. For some protein foods, such as legumes and cereals, there were considerable clinical trial data, but clinical data was limited (or non-existent) for algal, fresh fruit, and vegetable, insect, nut, and seed proteins. In these instances, animal model or in vitro study data was analysed and included in this review. A further layer of heterogeneity in the literature was the different forms of protein studied and the impact of different raising, cultivation, or processing protocols on health outcomes and bioactive efficacy. Processing also impacts health effects, with whole foods or protein concentrates being the least processed forms, while isolates provide the purest. Hydrolysates are generated by chemical or enzymatic digestion into smaller peptides, the products (and health effects) of which will vary depending on the process used. It is plausible that components other than protein (e.g., polyphenols, fibre) may contribute to observed health effects of the less processed forms. For many of the studies, control or comparator treatments consisted of dairy (whey, casein), soy protein, and, in some cases, meat. Protein digestibility data as measured by PDCAAS is available for a majority of the alternate protein foods, but most of this data is limited to the whole food and not its protein concentrate or isolate.

Data from human, animal, and in vitro studies suggest that alternative proteins may provide a range of health benefits that include glycaemic control, improved cardiovascular biomarkers, enhanced muscle synthesis (reduced sarcopenia), improved lipid metabolism, reduced protein malnutrition, and support a healthy gut microbiome (Table 1). Currently, there are no clinical trials reporting the health effects of algae, fruit, vegetable, or potato proteins; thus, we rely on preclinical studies to provide some insight. Animal and in vitro studies suggest that algae proteins may have a lower glycaemic response, contribute to appetite control, improved liver function (in NAFLD), and have antioxidant, anti-tumour, antihypertensive, and anti-inflammatory properties [2,3,7,8,9,10]. There were no studies that reported the health effects of proteins from fresh fruit, while data on protein from fresh vegetables was limited to potato. The consumption of potato protein showed a postprandial insulinemic response comparable to casein and whey protein [39]. It was also shown to augment the stimulatory effect of myofibrillar protein synthesis, both at rest and during exercise [41]. Possible palatability and sensory issues were noted, with protein isolates known to have a relatively better palatability profile compared to protein hydrolysates [42]. More studies are needed to compare physiological responses between different protein concentrates, isolates, and hydrolysates in humans.

Clinical results with cereal proteins from wheat (gluten), rice, and barley show either similar or improved health outcomes, when compared with traditional proteins. Results for glycogen synthesis with wheat protein hydrolysate are encouraging but inconsistent when studied in different cohorts [17,18]. The satiating effects of wheat protein are comparable with those of casein and whey [21]. Rice protein, when compared to whey, has similar effects on the body composition of exercising adults and was suggested to improve some inflammatory markers [24,25,26]. Animal studies with amaranth, quinoa, and buckwheat proteins suggest potential cardiovascular health benefits through reduced blood pressure, CRP, LDL cholesterol, and oxidative stress [29,30,31]. 

Insects have been consumed by humans for thousands of years, yet in developed countries neophobia, i.e., the ‘yuck’ factor, remains a major hurdle for food producers to overcome [134]. Of over 2000 species of edible insects [135], the industry is focusing on less than five species (mealworm, cricket, silkworm, termites, and grasshoppers). Only one human study was identified for cricket protein, showing a beneficial effect on the gut microbiome and a reduction in inflammatory cytokine TNF-α [49]. Animal and in vitro data show improved LDL cholesterol, antioxidant, anti-inflammatory, and antihypertensive activity [51,52,127]. A small human study of mealworm protein indicated more sustained protein digestion than whey isolate [48]. Studies in animals suggest a potential anti-obesity effect, improved lipid metabolism, cholesterol biosynthesis, and reduced plasma homocysteine [54,55,56,57]. Silkworm, likewise, shows a beneficial effect on hepatic steatosis and alcohol-induced fatty liver disease [63]. Termites are well-accepted in African communities, increasing the protein and mineral content of infant foods [47]. However, food allergy to insects has been described for some insect species [136]. Insect allergens are currently considered similar to shellfish allergies and labelled as such on products sold to consumers. Research is being conducted to identify insect allergens and the effect of food processing on their allergenicity, as well as to explore cost-effective and viable pathways to remove allergens [137].

Snail protein is also being used to supplement foods in developing countries, to prevent or treat malnutrition [65]. However, much more work is needed to identify optimal species for human protein consumption, optimise cultivation protocols, and conduct comprehensive, high quality human RCTs [137]. Cost and scalability are also key considerations for future insect and snail food ingredient production [138].

Human studies on protein from nuts and oil seeds are lacking. The focus of animal and in vitro studies has been on the antioxidant and antihypertensive properties of walnut protein [78,79,80,81,82,83]. Pine nut protein offers promise for type-2 diabetes with hypoglycaemic activity [88], while cashew nut protein peptides have exhibited antioxidant activity and cardiovascular benefits via the renin-angiotensin system [89]. Human studies with canola (rapeseed) protein show it to be as effective as soy protein regarding postprandial amino acid response and satiety [92,94]. Rat models indicate high digestibility and positive effects on hypertension [93]. More research is needed on the digestibility of complementary proteins from nuts and seeds, the role of anti-nutrients such as phytates, and the impact of cooking and processing on the health outcomes.

On the other hand, Mycoprotein is a non-traditional protein with considerable research interest for many years, with nine human studies identified. While these studies are mainly small in size and acute in scope, there is consistent evidence of health benefits. These include glycaemic control, myofibrillar protein synthesis and gene expression, total and LDL cholesterol reduction, satiety, and sustained plasma amino acid concentrations.

Although the health effects of soy proteins have been extensively investigated, only 22 human trials were identified for other legume proteins, such as pea and lupin. Pea protein was shown to reduce HbA1C in prediabetic adults [107], while a study comparing fava bean and pea protein in combination showed improved satiety compared to a high protein meat diet [118]. However, further studies using fibre-matched interventions saw no effect, suggesting the effect was likely due to non-protein components, such as fibre. Pea protein alone was also shown to improve satiety, although the effect seems to be dependent on the amount of protein, the gap between the protein meal and subsequent meals, and the presence of other food components such as fibre. Compared to animal protein, legume consumption modulated inflammatory markers including a reduction in CRP, IL6, and TNF-α [116,120]. The high arginine (Arg) content of lupin protein and the specific lupin protein γ- conglutin are suggested to contribute to the favourable changes in glycemic control and reduced LDL cholesterol, and improved LDL:HDL ratios, especially in hypercholesterolemic individuals following lupin consumption [117]. In support of these changes, one study showed that lupin reduced the plasma concentrations of PCSK9, an important enzyme involved in the regulation of lipid metabolism and cholesterol reduction [115]. Other components such as alkaloids in lupin likely contribute to the observed effect of lupin protein on LDL cholesterol since lupin protein isolates, as other plant protein isolates are seldom pure.

Algae, cricket, and mealworm proteins have been shown to elicit favourable changes in glycaemic control, glycated haemaglobin (HbA1c) levels, and/or improved postprandial insulinemia. Algae, cricket (protein hydrolysate), and mealworm protein (isolate and concentrate) have been associated with inhibition of dipeptidyl-peptidase IV (DPP-IV), an enzyme that inactivates incretins and gut-derived hormones. Inhibition of DPP-IV results in improved regulation of blood glucose levels and is currently a drug-based strategy that is used to manage type-2 diabetes [59]. Thus, these non-traditional proteins could offer a food-based approach to improve glycemic control through DPP-IV inhibitory activity.

Preclinical studies suggest that a range of non-traditional protein foods (barley, cricket, hemp seed, walnut, and snail) show potential in improving cardiovascular health through the inhibition of the angiotensin-I converting enzyme. This enzyme is important in the renin-angiotensin-aldosterone system, inhibition of which leads to vasodilation and reduced blood pressure, and is a large pharmaceutical market globally for hypertension and heart disease [139]. Importantly, these alternate proteins may offer a natural diet and non-drug-based approach to improve cardiovascular health if these findings can be confirmed in clinical trials.

Most non-traditional protein foods are plant-based (PB), bringing many synergistic factors with positive health benefits; phytoactive compounds with antioxidant, anti-inflammatory, and anti-carcinogenic activity. Aside from wheat gluten, complementary protein foods provide excellent alternatives for people with coeliac disease or gluten intolerance, as well as being lactose-free. Whole PB and insect foods provide dietary fibre and a diverse range of vitamins and minerals, providing beneficial health effects, alone or in combination with traditional protein sources. A further benefit in reducing animal protein intake is the lower intake of the amino acid methionine, which is associated with a protective effect against a range of cancers (and cancer recurrence), reduced DNA damage, and enhanced longevity [140].

Many of the findings presented here indicate that non-traditional protein sources offer compelling and beneficial health attributes, complementing traditional protein sources. However, in certain cases, potential risks or deleterious effects were reported. Rat data incorporating snail protein in diets, for example, resulted in a significant decrease in bone mineralization and mechanical strength [67]. Allergenicity and potential toxicity are also areas of a considerable research effort that were not in scope for this review but need to be considered, particularly in the case of algae and insect-derived proteins, as discussed above. Also of note is the potential for a change in micronutrient status as a result of ‘protein shift’ from predominantly animal-based to non-traditional protein consumption. Ongoing monitoring of important nutrients, such as iron, zinc, and vitamins B_12_ and D, will be required, particularly in developing countries and at-risk cohorts (women, children, and teenagers).

The authors note that a lack of preclinical and clinical evidence for the health benefits of non-traditional proteins is mainly due to this still being a relatively new area of research. Only one study (examining crickets) explored the effect of consumption on diversity of the human fecal microbiome. Thus, very little is known about the effect of complementary proteins on the gut microbiome, an area of considerable importance warranting further investigation. No studies have examined combinations of non-traditional and traditional proteins on health outcomes. Likewise, no studies were retrieved that examined the effect of complementary protein foods on cognitive function, mood, or depression. While limited, the data on inflammatory markers, cytokine production, and immune activation suggest this is an area in which non-traditional protein foods may have unique, positive health benefits. Considerable opportunity lies in expanding the evidence and exploring the effect of non-traditional proteins on the gut (and other) microbial communities, immune function, chronic inflammatory conditions, DNA damage, metabolomics, cognition, or cellular ageing. In addition to the effects of complementary proteins on individual biomarkers, epidemiological long-term evidence for whole diet approaches will be vitally important. Studies comparing the health of individuals who habitually consume either traditional or non-traditional protein foods over the long term will be of particular interest.

## 5. Conclusions and Future Directions

There is growing evidence that non-traditional, or complementary, dietary protein foods have great potential to enhance human health. For some complementary protein foods, the health effects are suggested to be specific to the protein component, while in other cases, components such as fibre or antioxidants may also contribute. More definitive effects of diverse proteins on health have been made when studies have involved protein concentrates, isolates, or hydrolysates, especially when directly compared with effects of meat, dairy, or soy protein consumption.

Complementary protein whole foods provide a diversity of both macro- and micronutrient components, together with dietary fibre and phytoactives with anti-inflammatory, anti-hypertensive, and antioxidant activity. The findings of this review also highlight opportunity for specific bioactive factors to be further explored as functional food components, or nutraceuticals, most notably for cardiovascular health, lipid metabolism, and glycaemic control.

The consistent theme across all protein sources examined in this review is that more well-designed, large-scale, long-term human intervention studies are needed. Detailed compositional analyses (particularly of different sub-species), food safety and toxicity, palatability, digestibility, and consumer acceptance studies will continue to be essential in the development of non-traditional dietary proteins. Advances in processing technologies, optimizing cultivation protocols, and improved sustainability practices will need to be developed in parallel with health and medical outcomes to deliver healthy foods for a growing global market. Quality, evidence-based health data is essential if complementary protein foods are to be shifted from ‘non-traditional’ to ‘typical/conventional’, and to facilitate development of high-quality protein foods that enhance human health for all stages of life.

## Figures and Tables

**Table 1:** Non-traditional protein sources: Summary of health effects from human, animal, and in vitro studies.

Protein Source	Human Studies (#)	PDCAAS * (In Vitro)	Potential Health Effects	Knowledge Gaps and Future Directions
Algae	0	0.29–0.64 [121]	Improved glycaemic status Appetite control Improved liver function in NAFLD Modulate antioxidant, anti-inflammatory and ACE inhibition actions for disease prevention	Human clinical and protein bioavailability data needed Optimised algal growth conditions Protein extraction protocols Safety & toxicity testing Testing and selection of optimal species for human consumption
Cereal, Barley	1	0.59–0.76 [122,123]	Comparable with casein with respect LDL cholesterol, inflammation (CRP), oxidative stress and blood pressure	More human clinical data needed to determine whether effects are specific to barley as a whole food or protein fraction.
Cereal, Buckwheat	1	0.041–0.5 [123]	Lipid profile and inflammatory markers improved in a cohort with mild/moderate hypercholesterolemia	More human clinical data needed to determine whether effects are specific to whole food or protein fraction.
Cereal, Oat	1	0.67 [124]	Hunger/appetite suppression Increased plasma insulin	More human clinical data needed to determine whether effects are specific to whole food or protein fraction.
Cereal, Rice	7	0.51–0.62 [124]	Reduced pro-inflammatory cytokines. Compares well with whey for body composition with exercise	Further analysis needed for different processing methods and
Cereal, Rye	1	0.59 [122]	Improved satiety Improved biomarkers of glycaemic control	More human clinical data needed
Cereal, Wheat	7	0.42–0.54 [123,125]	Increased glycogen synthesis Supported exercise and muscle performance and reduced exercise-induced inflammation Myofibrillar protein synthesis lower than whey or casein Improve blood lipid profile and anti-hypertensive effects Assist with energy balance and improve satiety Elicits insulin response	More human clinical data needed including myofibrillar synthesis and satiety Greater clarity whether effects are due to protein alone, or whole food
Fresh fruit	0	n/a	No studies identified	
Fresh vegetable, potato	4	0.87–1.0 [123]	Potato protein augments effect of myofibrillar protein synthesis Increased glucose control	More human clinical data needed
Insect, Cricket (ground) *Gryllus assimilis*	1	0.65–0.73 [126,127]	Improved gut microbiome Reduced inflammation Reduced LDL cholesterol Bioactives (antioxidants, anti-inflammatories, ACE inhibition, DPP-IV inhibition)	Human clinical data needed Scalability and consistency of production Production cost Overcoming the ‘yuck’ factor
Insect, Mealworm (ground) *Tenebrio molitor*	1	0.54 [126]	Slower, sustained amino acid digestion Improved glucose tolerance Improved lipid metabolism Potential anti-obesity effects Reduced homocysteine Bioactives (antioxidants, anti-inflammatories, ACE inhibition, DPP-IV inhibition)	Human clinical data needed Scalability and consistency of production Production cost Overcoming the ‘yuck’ factor
Silkworm *Bombyx mori*	0	N/A for *B. mori* (*Samia ricinii* 0.86 [128])	Improved lipid metabolism Improved fatty liver disease Anti-inflammatory factors	Human clinical data needed Scalability and consistency of production
Termites *Macrotermes* *nigeriensis*	2	0.42 [127]	Rich in protein Rich in minerals (Mg, Ca, K, P) Well tolerated and accepted for infant food supplementation	Human clinical data needed Scalability and consistency of production
Snails	0	N/A	Improved glycaemic control and diabetic complications Improved malnutrition Bioactives, including ACE inhibitor Deleterious effect on bone mineralisation and strength	Human clinical data needed Scalability and consistency of production More bone health studies needed
Myco- protein	9	1.0 [129]	Improved myofibrillar protein synthesis and gene expression Stimulated post exercise tissue remodelling Comparable to milk, fish and meat protein for glycaemic control Improved satiety Improved total and LDL cholesterol, lipid metabolism Slower, sustained amino acid release	Larger cohorts needed to confirm effects
Nuts	0	0.22 (almonds) 0.81 (roasted pistachios) (rat bioassay) [130]	Improved cognition and memory (walnuts, pine nuts) Bioactives (antioxidant, antihypertensive, antiinflammation)	Human clinical data needed. Low-moderate PDCAAS scores.
Oil Seeds	3	0.5–0.6 (hemp seed) (rat bioassay) [131]	Improved hypoglycaemic response (canola/rapeseed) Improved hypotensive response (hemp seed, sesame seeds) Improved satiety Improved cholesterol (sesame seed) Improved antioxidant capacity	More human studies needed.
Legumes, Beans	3 (in mixed legume diet)	Fava bean 0.56 [126] Cooked beans 0.54–0.75 Extruded beans 0.58–0.69 Baked beans 0.47–0.66 ** [132]	Improved satiety Reduced inflammatory cytokines (CRP, IL6, TNFα)	Specific clinical studies on bean proteins not available. More human studies required
Legumes, Peas	14	Yellow pea 0.59 [126] Cooked (0.69–0.72) Extruded (0.65–0.73) Baked (0.69–0.75) [132]	Improved satiety Reduced postprandial diastolic blood pressure (pea protein isolate) and systolic blood pressure in longer term (hydrolysate) Reduced postprandial blood glucose and HbA1c (hydrolysate) Reduced inflammatory cytokines (CRP, IL6, TNFα)	Lack of consistency in satiety and blood glucose outcomes. More studies required to confirm effects on blood pressure and inflammatory biomarkers
Legumes, Lentils	3	0.68–0.80 [123]	Improved satiety Reduced postprandial blood glucose Heme-iron absorption maintained Reduced inflammatory cytokines (CRP, IL6, TNFα)	Limited clinical data available on lentil proteins
Legumes, Chickpeas	1	0.69–0.77 [123]	Reduced inflammatory cytokines (CRP, IL6, TNFα)	No clinical data on purified chickpea proteins
Legumes, Lupin	7	0.6 [133]	Improved hyperglycaemia (conglutin) Reduced LDL cholesterol and LDL:HDL ratio, especially in hypercholesterolemic subjects Reduced PCSK9 expression (improve lipid and cholesterol management) Reduced inflammatory cytokines and Th1-cell activation Increased antioxidant capacity of PBMCs	Insufficient clinical data available on glycaemic and immune responses

* Protein Digestibility Corrected Amino Acid Score (PDCAAS) is a method of evaluating the quality of a protein based on both the amino acid requirements of humans and their ability to digest it. ** Values dependent on variety; (ACE, angiotensin-I converting enzyme; CRP, C-reactive protein; DPP-IV, dipeptidyl-peptidase 4; HbA1c, glycated haemoglobin; HDL, high density lipoprotein; IL6, interleukin 6; LDL, low density lipoprotein; NAFLD, non-alcoholic fatty liver disease; PBMCs, peripheral blood mononuclear cells; PCSK9, Proprotein convertase subtilisin/kexin type 9; TNF, tumor necrosis factor).

## Data Availability

No new data were created or analyzed in this study. Data sharing is not applicable to this article.

## Supplementary Materials

The following are available online at https://www.mdpi.com/article/10.3390/foods11040528/s1, Table S1a,b: Algae, Human Studies (Not specific to algal protein) & Algae Protein, Animal Studies, Table S2: Cereal Proteins, Human Studies, Table S3: Fresh Fruits and Vegetable Protein, Human Studies; Table S4: Insect and Snail Protein, Human, Animal and In vitro Studies; Table S5: Mycoprotein, Human Studies; TableS 6: Nut and Oil Seed Protein, Human, Animal and In vitro Studies; Table S7: Legume (non-soy) Protein, Human Studies.

## Appendix A. Search Strings

PubMed Search Strategy

(“Diet, food, and nutrition”[MeSH Terms] OR diet[Title/Abstract] OR food[Title/Abstract] OR nutrition[Title/Abstract]) AND (“Insect Proteins”[MeSH Terms] OR “grain proteins”[MeSH Terms] OR “seed storage proteins”[MeSH Terms] OR “plant proteins, dietary”[MeSH Terms] OR “dietary proteins”[MeSH Terms] OR protein[Title/Abstract] OR proteins[Title/Abstract] OR protein’s[Title/Abstract]) AND (“Insecta”[MeSh Terms] OR “Weevils”[MeSH Terms] OR weevil[Title/Abstract] OR weevils[Title/Abstract] OR Stratiomyidae[Title/Abstract] OR soldier fly[Title/Abstract] OR soldier flies[Title/Abstract] OR “Grasshoppers”[MeSH Terms] OR grasshopper[Title/Abstract] OR grasshoppers[Title/Abstract] OR locust[Title/Abstract] OR locusts[Title/Abstract] OR “Odonata”[MeSH Terms] OR Odonata[Title/Abstract] OR dragonfly[Title/Abstract] OR dragonflies[Title/Abstract] OR “Ants”[MeSH Terms] OR ant[Title/Abstract] OR ants[Title/Abstract] OR “Isoptera”[MeSH Terms] OR Isoptera[Title/Abstract] OR termite[Title/Abstract] OR termites[Title/Abstract] OR “Moths”[MeSH Terms] OR bogong moth[Title/Abstract] OR bogong moth[Title/Abstract] OR Bombyx[Title/Abstract] OR silkworm[Title/Abstract] OR silkworms[Title/Abstract] OR “Coleoptera”[MeSH] OR Coleoptera[Title/Abstract] OR beetle[Title/Abstract] OR beetles[Title/Abstract] OR superworm[Title/Abstract] OR superworm[Title/Abstract] OR superworms[Title/Abstract] OR Zophobas[Title/Abstract] OR king worm[Title/Abstract] OR king worms[Title/Abstract] OR morio worm[Title/Abstract] OR morio worms[Title/Abstract] OR “Tenebrio”[MeSH] OR Tenebrio[Title/Abstract] OR mealworm[Title/Abstract] OR mealworms[Title/Abstract] OR “Gryllidae”[MeSH Terms] OR Gryllidae[Title/Abstract] OR cricket[Title/Abstract] OR crickets[Title/Abstract] OR “Snails”[MeSH Terms] OR snail[Title/Abstract] OR snails[Title/Abstract] OR “Yeast, Dried”[MeSH Terms] OR yeast[Title/Abstract] OR “Acacia”[MeSH Terms] OR Acacia[Title/Abstract] OR wattle[Title/Abstract] OR wattleseed[Title/Abstract] OR “Lycopersicon esculentum”[MeSH Terms] OR Lycopersicon esculentum[Title/Abstract] OR tomato[Title/Abstract] OR tomatoes[Title/Abstract] OR “Spinacia oleracea”[MeSH Terms] OR Spinacia oleracea[Title/Abstract] OR spinach[Title/Abstract] OR “Manihot”[MeSH Terms] OR Manihot[Title/Abstract] OR cassava[Title/Abstract] OR “Nuts”[MeSH Terms] OR nut[Title/Abstract] OR nuts[Title/Abstract] OR “Prunus dulcis”[MeSH Terms] OR Prunus dulcis[Title/Abstract] OR almond[Title/Abstract] OR almonds[Title/Abstract] OR “Bertholletia”[MeSH Terms] OR Bertholletia[Title/Abstract] OR brazil nut[Title/Abstract] OR brazil nuts[Title/Abstract] OR “Anacardium”[MeSH Terms] OR Anarcardium[Title/Abstract] OR cashews[Title/Abstract] OR “Corylus”[MeSH Terms] OR Corylus[Title/Abstract] OR hazelnut[Title/Abstract] OR hazelnuts[Title/Abstract] OR “Arachis”[MeSH Terms] OR Arachis[Title/Abstract] OR peanut[Title/Abstract] OR peanuts[Title/Abstract] OR “Pistacia”[MeSH Terms] OR Pistacia[Title/Abstract] OR pistachio[Title/Abstract] OR pistachios[Title/Abstract] OR “Juglans”[MeSh Terms] OR Juglans[Title/Abstract] OR walnut[Title/Abstract] OR walnuts[Title/Abstract] OR “Sesamum”[MeSH Terms] OR Sesamum[Title/Abstract] OR sesame[Title/Abstract] “Chrysophyta”[MeSH Terms] OR Chrysophyta[Title/Abstract] OR “Rhodophyta”[MeSH Terms] OR Rhodophyta[Title/Abstract] OR “Phaeophyta”[MeSH Terms] OR Phaeophyta[Title/Abstract] OR “Chlorophyta”[MeSH Terms] OR Chlorophyta[Title/Abstract] OR “Microalgae”[MeSH Terms] OR algae[Title/Abstract] OR macroalgae[Title/Abstract] OR microalgae[Title/Abstract] OR “Seaweed”[MeSH Terms] OR seaweed[Title/Abstract] OR “Spirulina”[MeSH Terms] OR spirulina[Title/Abstract] OR chlorella[Title/Abstract] OR “Amaranthus”[MeSH Terms] OR Amaranthus[Title/Abstract] OR amaranth[Title/Abstract] OR “Eragrostis”[MeSH Terms] OR Eragrostis[Title/Abstract] OR teff[Title/Abstract] OR “Sorghum”[MeSH Terms] OR sorghum[Title/Abstract] OR “Secale”[MeSH Terms] OR Secale[Title/Abstract] OR rye[Title/Abstract] OR “Hordeum”[MeSH Terms] OR Hordeum[Title/Abstract] OR barley[Title/Abstract] OR “Zea mays”[MeSH Terms] OR Zea mays[Title/Abstract] OR maize[Title/Abstract] OR corn[Title/Abstract] OR “Triticum”[MeSH Terms] OR Triticum[Title/Abstract] OR wheat[Title/Abstract] OR freekeh[Title/Abstract] OR “peas”[MeSH Terms] OR peas[Title/Abstract] OR pea[Title/Abstract] OR “pisum sativum”[Title/Abstract] OR “lens plant”[MeSH Terms] OR “lens plant*”[Title/Abstract] OR lentil[Title/Abstract] OR lentils[Title/Abstract] OR lens culinaris[Title/Abstract] OR “cicer”[MeSH Terms] OR cicer[Title/Abstract] OR chickpea[Title/Abstract] OR chickpea’[Title/Abstract] OR chickpea’s[Title/Abstract] OR chickpeas[Title/Abstract] OR chickpeas’[Title/Abstract] OR garbanzo[Title/Abstract] OR “vicia faba”[MeSH Terms] OR vicia faba[Title/Abstract] OR faba bean[Title/Abstract] OR faba beans[Title/Abstract] OR fava bean[Title/Abstract] OR fava beans[Title/Abstract] OR broad bean[Title/Abstract] OR broad beans[Title/Abstract] OR “helianthus”[MeSH Terms] OR helianthus[Title/Abstract] OR sunflower[Title/Abstract] OR sunflower’[Title/Abstract] OR sunflower’s[Title/Abstract] OR sunflowers[Title/Abstract] OR sunflowerseed[Title/Abstract] OR sunflowerseeds[Title/Abstract] OR “cucurbita”[MeSH Terms] OR cucurbita[Title/Abstract] OR pumpkin[Title/Abstract] OR pumpkin’[Title/Abstract] OR pumpkin’s[Title/Abstract] OR pumpkins[Title/Abstract] OR pumpkinseed[Title/Abstract] OR pumpkinseed’s[Title/Abstract] OR pumpkinseeds[Title/Abstract] OR “fagopyrum”[MeSH Terms] OR fagopyrum[Title/Abstract] OR buckwheat[Title/Abstract] OR “chenopodium quinoa”[MeSH Terms] OR “chenopodium quinoa”[Title/Abstract] OR quinoa[Title/Abstract] OR “solanum tuberosum”[MeSH Terms] OR Potato[Title/Abstract] OR potatoes[Title/Abstract] OR “solanum tuberosum”[Title/Abstract] OR mushroom[Title/Abstract] OR mushrooms[Title/Abstract] OR mycoprotein[Title/Abstract] OR “oryza”[MeSH Terms] OR “oryza sativa”[Title/Abstract] OR rice[Title/Abstract] OR “lupinus”[MeSH Terms] OR lupinus[Title/Abstract] OR lupin[Title/Abstract] OR lupins[Title/Abstract] OR “avena”[MeSH Terms] OR “avena sativa”[Title/Abstract] OR oat[Title/Abstract] OR oats[Title/Abstract] OR duckweed[Title/Abstract] OR wolffia[Title/Abstract] OR mankai[Title/Abstract] OR lemnaceae[Title/Abstract] OR lemna[Title/Abstract] OR lemnoideae[Title/Abstract] OR “medicago sativa”[MeSH Terms] OR “medicago sativa”[Title/Abstract] OR alfalfa[Title/Abstract] OR plukenetia volubilis[Title/Abstract] OR sacha inchi[Title/Abstract] OR “cannabis”[MeSH Terms] OR Hemp[Title/Abstract] OR cannabis[Title/Abstract] OR rapeseed*[Title/Abstract] OR “Brassica”[MeSH Terms] OR brassica[Title/Abstract] OR rape seed[Title/Abstract] OR canola[Title/Abstract]) AND (Human health[Title/Abstract] OR disease risk[Title/Abstract] OR health effect[Title/Abstract] OR health effects[Title/Abstract] OR health promoting[Title/Abstract] OR health-promoting[Title/Abstract] OR health impact[Title/Abstract] OR health impacts[Title/Abstract] OR health benefit[Title/Abstract] OR nutritive value[Title/Abstract] OR “Inflammation”[MeSH Terms] OR inflammation[Title/Abstract] OR inflammatory[Title/Abstract] OR allergenicity[Title/Abstract] OR “appetite”[MeSH Terms] OR appetite[Title/Abstract] OR appetite regulation[Title/Abstract] OR “satiety response”[MeSH Terms] OR satiety[Title/Abstract] OR satiation[Title/Abstract] OR “hunger”[MeSH Terms] OR hunger[Title/Abstract] OR fullness[Title/Abstract] OR “cholecystokinin”[MeSH Terms] OR cholecystokinin[Title/Abstract] OR CCK[Title/Abstract] OR “leptin”[MeSH Terms] OR leptin[Title/Abstract] OR “glucagon like peptide 1”[MeSH Terms] OR “glucagon-like peptide-1”[Title/Abstract] OR GLP-1[Title/Abstract] OR “peptide yy”[MeSH Terms] OR “peptide yy”[Title/Abstract] OR “ghrelin”[MeSH Terms] OR ghrelin[Title/Abstract] OR “visual analog scale”[MeSH Terms] OR “visual analog scale”[Title/Abstract] OR “postprandial glycemia”[Title/Abstract] OR “postprandial glycaemia”[Title/Abstract] OR “visual analogue scale”[Title/Abstract] OR “body weight”[MeSH Terms] OR “body weight”[Title/Abstract] OR “body weight changes”[Title/Abstract] OR “body weight changes”[MeSH Terms] OR “body mass index”[MeSH Terms] OR “body mass index”[Title/Abstract] OR “fat free mass”[Title/Abstract] OR “fat-free mass”[Title/Abstract] OR “weight loss”[MeSH Terms] OR “weight loss”[Title/Abstract] OR “weight reduction”[Title/Abstract] OR “body composition”[MeSH Terms] OR “body composition”[Title/Abstract] OR “waist circumference”[MeSH Terms] OR “waist circumference”[Title/Abstract] OR “abdominal fat”[MeSH Terms] OR “abdominal fat”[Title/Abstract] OR “visceral fat”[Title/Abstract] OR “body fat”[Title/Abstract] OR “abdominal visceral fat”[Title/Abstract] OR “adipose tissue”[MeSH Terms] OR “adipose tissue”[Title/Abstract] OR “adiposity”[MeSH Terms] OR adiposity[Title/Abstract] OR “overweight”[MeSH Terms] OR overweight[Title/Abstract] OR “obesity”[MeSH Terms] OR obesity[Title/Abstract] OR obese[Title/Abstract] OR “diabetes mellitus”[MeSH Terms] OR diabetes[Title/Abstract] OR diabetic[Title/Abstract] OR “hyperglycemia”[MeSH Terms] OR hyperglycemia[Title/Abstract] OR hyperglycemic[Title/Abstract] OR glycemic response[Title/Abstract] OR glycaemic response[Title/Abstract] OR “blood glucose”[MeSH Terms] OR “blood glucose”[Title/Abstract] OR “insulin”[MeSH Terms] OR insulin[Title/Abstract] OR “insulin resistance”[MeSH Terms] OR “postprandial glucose”[Title/Abstract] OR “postprandial insulin”[Title/Abstract] OR “fasting glucose”[Title/Abstract] OR f-glc[Title/Abstract] OR “homeostasis model assessment-insulin resistance”[Title/Abstract] OR HOMA-IR[Title/Abstract] OR HOMA-IS[Title/Abstract] OR “glucose tolerance test”[MeSH Terms] OR “glucose tolerance test”[Title/Abstract] OR OGTT[Title/Abstract] OR “AUC glucose”[Title/Abstract] OR “iAUC glucose”[Title/Abstract] OR “AUC insulin”[Title/Abstract] OR “iAUC insulin”[Title/Abstract] OR “cardiovascular diseases”[MeSH Terms] OR “cardiovascular diseases”[Title/Abstract] OR “cardiovascular disease”[Title/Abstract] OR “hypercholesterolemia”[MeSH Terms] OR hypercholesterolemia[Title/Abstract] OR hypercholesterolemic[Title/Abstract] OR “hyperlipidemias”[MeSH Terms] OR hyperlipidemia[Title/Abstract] OR hyperlipidemias[Title/Abstract] OR hyperlipidemic[Title/Abstract] OR “dyslipidemias”[MeSH Terms] OR dyslipidemia[Title/Abstract] OR dyslipidemias[Title/Abstract] OR dyslipidemic[Title/Abstract] OR “blood lipid”[Title/Abstract] OR “blood lipids”[Title/Abstract] OR “lipid profile”[Title/Abstract] OR “cholesterol”[MeSH Terms] OR cholesterol[Title/Abstract] OR “triglycerides”[MeSH Terms] OR triglyceride[Title/Abstract] OR triglycerides[Title/Abstract] OR trigliceride[Title/Abstract] OR triglicerides[Title/Abstract] OR HDL[Title/Abstract] OR LDL[Title/Abstract] OR “metabolic syndrome”[MeSH Terms] OR “metabolic syndrome”[Title/Abstract] OR “Metabolome”[MeSH Terms] OR Metabolome[Title/Abstract] OR “blood pressure”[MeSH Terms] OR “blood pressure”[All Fields] OR systolic[Title/Abstract] OR diastolic[Title/Abstract] OR “hypertension”[MeSH Terms] OR hypertension[Title/Abstract] OR hypertensive[Title/Abstract] OR antihypertensive[Title/Abstract] OR “muscles”[MeSH Terms] OR muscle[Title/Abstract] OR muscles[Title/Abstract] OR “Sarcopenia”[MeSH Terms] OR Sarcopenia[Title/Abstract] OR “Frailty”[MeSH Terms] OR frailty[Title/Abstract] OR renal function[Title/Abstract] OR “Bone and Bones”[MeSH Terms] OR bone health[Title/Abstract] OR bone density[Title/Abstract] OR bone mineralisation[Title/Abstract] OR musculoskeletal health[Title/Abstract] OR genetic toxicology[Title/Abstract] OR “DNA Damage”[MeSH Terms] OR DNA Damage[Title/Abstract] OR DNA health[Title/Abstract] OR “Telomere”[MeSH Terms] OR Telomere length[Title/Abstract] OR “Gastrointestinal Microbiome”[MeSH Terms] OR gastrointestinal microbiome[Title/Abstract] OR gut microbiome[Title/Abstract] OR gastrointestinal microbiota[Title/Abstract] OR gut microbiome[Title/Abstract] OR gut health[Title/Abstract] OR “Cognition”[MeSH Terms] OR cognition[Title/Abstract] OR cognitive ability[Title/Abstract] OR “Affect”[MeSH Terms] OR affect[Title/Abstract] OR mood[Title/Abstract] OR brain health[Title/Abstract] OR “Healthy Aging”[MeSH Terms] OR healthy aging[Title/Abstract] OR “neoplasms”[MeSH Terms] OR cancer[tiab] OR cancers[tiab] OR carcinoma[tiab] OR carcinomas[tiab] OR neoplasm[tiab] OR neoplasms[tiab] OR tumor[tiab] OR tumors[tiab] OR tumour[tiab] OR tumours[tiab]) AND (randomized controlled trial [pt] OR controlled clinical trial [pt] OR randomized [tiab] OR placebo [tiab] OR drug therapy [subheading] OR randomly [tiab] OR trial [tiab] OR groups [tiab]) NOT (“animals”[MeSH Terms] NOT “humans”[MeSH Terms]).

Web of Science Core Collection Strategy

TS = (Diet OR food OR nutrition) AND TS = ((Protein OR proteins OR protein’s) NEAR/5 (weevil OR Stratiomyidae OR “soldier fly” OR “soldier flies”OR Grasshopper* OR locust OR locusts OR Odonata OR dragonfly OR dragonflies OR ant OR ants OR Isoptera OR termite* OR “bogong moth*” OR Bombyx OR silkworm* OR Coleoptera OR beetle OR beetles OR superworm* OR Zophobas OR “king worm*” OR “morio worm*” OR Tenebrio OR mealworm* OR Gryllidae OR cricket* OR snail* OR yeast OR Acacia OR wattle OR wattleseed OR “Lycopersicon esculentum” OR tomato* OR “Spinacia oleracea” OR spinach OR Manihot OR cassava OR nut OR nuts OR “Prunus dulcis” OR almond* OR Bertholletia OR “brazil nut*” OR Anarcardium OR cashews OR Corylus OR hazelnut* OR Arachis OR peanut* OR Pistacia OR pistachio* OR Juglans OR walnut* OR Sesamum OR sesame OR Chrysophyta OR Rhodophyta OR Phaeophyta OR Chlorophyta OR algae OR macroalgae OR microalgae OR seaweed OR spirulina OR chlorella OR Amaranthus OR amaranth OR Eragrostis OR teff OR sorghum OR Secale OR rye OR Hordeum OR barley OR “Zea mays” OR maize OR corn OR Triticum OR wheat OR freekeh OR pea OR peas OR “pisum sativum” OR “lens plant*” OR lentil OR lentils OR “lens culinaris” OR cicer OR chickpea* OR garbanzo OR “vicia faba” OR “faba bean*” OR “fava bean*” OR “broad bean*” OR helianthus OR sunflower* OR cucurbita OR pumpkin* OR fagopyrum OR buckwheat OR quinoa OR “solanum tuberosum” OR Potato* OR mushroom* OR mycoprotein OR “oryza sativa” OR rice OR lupin* OR “avena sativa” OR oat* OR duckweed OR wolffia OR mankai OR lemna* OR lemnoideae OR “medicago sativa” OR alfalfa OR “plukentetia volubilis” OR “sacha inchi” OR hemp OR cannabis OR rapeseed OR brassica OR “rape seed” OR canola)) AND TS = (“Human health” OR “disease risk” OR “health effect*” OR “health promoting” OR health-promoting OR “health impact*” OR “health benefit” OR “nutritive value” OR inflammation OR inflammatory OR allergenicity OR appetite OR satiety OR satiation OR hunger OR fullness OR cholecystokinin OR CCK OR leptin OR “glucagon like peptide 1” OR “glucagon-like peptide-1” OR GLP-1 OR “peptide yy” OR “peptide yy” OR ghrelin OR “visual analog scale” OR “postprandial glycaemia” OR “visual analogue scale” OR “body weight” OR bodyweight OR “body mass index” OR BMI OR “fat free mass” OR “fat-free mass” OR “weight loss” OR “weight reduction” OR “body composition” OR “waist circumference” OR “abdominal fat” OR “visceral fat” OR “body fat” OR “abdominal visceral fat” OR “adipose tissue” OR adiposity OR overweight OR obesity OR obese OR diabetes OR diabetic OR hyperglycemia OR hyperglycemic OR “glycemic response” OR “glycaemic response” OR “blood glucose” OR insulin OR “postprandial glucose” OR “postprandial insulin” OR “fasting glucose” OR f-glc OR “homeostasis model assessment-insulin resistance” OR HOMA-IR OR HOMA-IS OR “glucose tolerance test” OR OGTT OR “AUC glucose” OR “iAUC glucose” OR “AUC insulin” OR “iAUC insulin” OR “cardiovascular disease*” OR hypercholesterolemia* OR hyperlipidemi* OR dyslipidemi* OR “blood lipids” OR “lipid profile” OR cholesterol OR triglyceride* OR triglyceride* OR HDL OR LDL OR “metabolic syndrome” OR Metabolome OR “blood pressure”OR systolic OR diastolic OR hypertens* OR antihypertensive OR muscle* OR Sarcopenia OR frailty OR “renal function” OR “bone health” OR “bone density” OR “bone mineralisation” OR “musculoskeletal health” OR “genetic toxicology” OR “DNA Damage” OR “DNA health” OR Telomere OR “Gastrointestinal Microbiome” OR “gut microbiome” OR “gastrointestinal microbiota” OR “gut microbiome” OR “gut health” OR cognition OR “cognitive ability” OR affect OR mood OR “brain health” OR “healthy aging” OR neoplasm* OR cancer* OR carcinoma* OR tumor* OR tumour*) AND TS = (“clinical trial*” OR “research design” OR “comparative stud*” OR “evaluation stud*” OR “controlled trial*” OR “follow-up stud*” OR “prospective stud*” OR random* OR placebo* OR “single blind* OR “double blind*”).
