# Supplementary material for: How Healthy Are Non-Traditional Dietary Proteins? The Effect of Diverse Protein Foods on Biomarkers of Human Health
Source: Foods. 2022 Feb 11;11(4):528. doi: 10.3390/foods11040528 (PMC8871094; doi:10.3390/foods11040528)
Supplement: Supplementary file 1 [file foods-11-00528-s001.zip › foods-1549600-supplementary.pdf]

Supplementary Table S1a. Algae, Human Studies (Not specific to algal protein)

Protein type & form	Biomarker / Health measures	Reference	Study type	Study summary	Outcome
Two brown seaweeds: Laminaria digitata (LD) Undaria pinnatifida (UP)	Glycemic and insulinemic responses Glucagon-like peptide-1 (GLP-1) secretion Appetite	Zaharudin et al 2021	Human RCT N = 20 healthy males and females aged 20-50 y	Compared Fasting and postprandial blood test after consuming one of three meals comprising 30 g of starch with 5 g of LD or UP or an energy-adjusted control meal containing pea protein at each test day.	A lower blood glucose, insulin and C-peptide response following the intake of LD and UP Concomitant ingestion of brown seaweeds may help improving postprandial glycaemic and appetite control in healthy and normal weight adults.
Aquamin® product obtained from the skeletal remains of red marine algae of the Lithothamnion genus	Abundance of protein markers including Ki67 (proliferation marker) and CK20 and p21 (differentiation markers) Whole proteome analysis	Aslam et al 2021	Human RCT N = 30 healthy aged 18-80 y, male or female (at risk for colorectal cancer)	Compared Aquamin® to calcium alone and placebo in a 90-day interventional trial. Before and after the interventional period, colonic biopsies were obtained The effects of Aquamin® or calcium alone on protein profile in the colonic mucosa were analysed using proteomics.	Daily Aquamin® ingestion alters protein abundance profile in the colon that could be beneficial to colonic health. Along with higher alterations in abundance of proteins in the treatment with Aquamin® than calcium, the reduced abundance of Ki67 marker was also identified.
Tropical green seaweeds: Caulerpa racemosa and Caulerpa scalpelliformis Brown seaweeds: Spatoglossum asperum and Sargassum linearifolium	Anti-proliferative ROS inhibitory activities including transcript expression of cancer-linked key genes and apoptosis	Tanna et al 2020	In vitro HeLa and Huh-7 cells	Evaluation of cancer preventive, and nutraceutical properties of tropical seaweeds	Caulerpa spp. are rich in antioxidant and anti-proliferative activities. The extracts from Caulerpa spp. regulate the transcript levels of key cancer-linked marker genes Caulerpa spp. have potential to be explored further for cancer preventive properties or functional food or nutraceuticals applications.
Microalgae including: Chlorella vulgaris	Gut microbiota Colonic fermentation of microalgae Bioaccessibility of algae	Jin et al 2020	In vitro	Simulated digestion and colonic fermentation were examined to investigate the effects of microalgae samples on gut microbiota.	The study suggests the potential use of microalgae as a functional food to increase propionate generation because propionate has been reported to be effective in weight loss and the inhibition of pathogen infection.

Chlorella protothecoides Schizochytrium sp .					Following in vitro digestion, the protein content of chlorella groups notably decreased, showing that the high bioaccessibility of them might be due to its protein being sufficiently digested during the in vitro digestion process. The protein content in the chlorella groups was still high after the simulated digestion compared to Schizochytrium sp. group.
ACE inhibitory peptides (ACEIPs) from the protein hydrolysate of the marine macroalga Ulva intestinalis	ACE Inhibitory Peptides In Vitro Digestion of the ACE peptides	Sun et al 2019	In Vitro	ACE inhibitory peptides were isolated and purified from Ulva intestinalis using different proteases and the molecular mass and amino acid sequence of them were identified.	Two novel purified ACE inhibitors were identified. Based on in vitro digestion results, both peptides demonstrated good stability for pepsin and trypsin digestion. This study suggested the high-valued application of U. intestinalis and the development of food-derived ACE inhibitory peptides.
Aqueous high molecular weight sulfated polysaccharides (SPs) extracts of green algae, Ulva lactuca	Antioxidant Activity Antitumor Activity	Abou El Azm et al 2019	In vitro	Post-Extraction Enzymatic Hydrolysis Followed by Ion-Exchange Chromatographic Fractionation was used to examine the carbohydrate, protein, and sulfate contents in eight different molecular weight fractions resulted from the fractionation method. The results of the in vitro analysis of antioxidant and antitumor activities of different fractions were compared and correlated with the results obtained from their molecular contents.	The antioxidant and antitumor activities are influenced by various factors in algae extracts including their protein content. The fractions with highest in protein content showed antioxidant activity above the 60%, so it could be attributed to the higher amount of protein, which indicates higher content of amide groups. The proposed process combining enzymatic hydrolysis and ion-exchange chromatography could potentially be used in food and pharmaceutical industries as a post extraction step to produce compounds with better bioactivities than the polysaccharide extracts.
Brown seaweed (Ascophyllum nodosum)	Bioavailability DNA damage Oxidative stress Inflammation C-reactive protein (CRP)	Baldrick et al 2018	Human RCT N = 80 aged 30–65 y urine and plasma samples	Metabolomics analysis of urine and plasma samples after algae consumption. Daily consumption of either a 400-mg capsule containing 100 mg seaweed (poly)phenol and 300 mg maltodextrin or a 400-mg maltodextrin placebo control capsule for an 8-wk period.	Consumption of the seaweed (poly)phenols resulted in a modest decrease in DNA damage but only in a subset of the total population who were obese.
Ulva compressa Ulva rigida Gelidium microdon	I-converting enzyme (ACE) inhibitory properties Nutrient analysis	Paiva et al 2017	In vitro	Biochemical composition and in vitro algae protein digestibility	Protein was one of the most abundant components in red and green macroalgae All the macroalgal proteins had high digestibility in vitro and contained high quantity of essential amino acids

Pterocladiella capillacea	In vitro algae protein digestibility				ACE-inhibitory peptide fractions, particularly from Ulva species, might have a potential use as an ingredient for antihypertensive functional foods and nutraceuticals formulation
Chlorella vulgaris	Inflammatory biomarkers in patients with Non-alcoholic fatty liver disease (NAFLD)<br>Glucose homeostasis<br>Insulin resistance	Ebrahimi-Mameghani et al 2017	Human RCT<br>N = 70 NAFLD patients	Compared intervention group (four 300 mg tablets of C. vulgaris) or placebo group (four 300 mg tablets of placebos) for 8 weeks.<br>Compared plasma samples before and after the intervention.	C. vulgaris supplementation could be considered as an adjunctive therapy to decrease weight and improve glycemic status and reducing hs-CRP as well as improving liver function in patients with NAFLD.
Palmaria palmata	Serum markers of inflammation [C-reactive protein (CRP); cytokine analysis]<br>Changes in lipids (cholesterol, triglycerides),<br>Thyroid function [thyroid-stimulating hormone (TSH)]<br>Oxidative stress	Allsopp et al 2016	Human RCT<br>N = 40, aged 18–65 y<br>And In vitro (using a Caco-2 inflammation cell model)	Compared consuming breads containing p. palmata at 5 g/d (treatment group) and without p. palmata (control group) for 4 weeks.<br>In vitro studies on inflammatory activity of P. Palmata extracts, protein extracts and associated protein hydrolysates using a Caco-2 inflammation cell model.	Consumption of the P. palmata bread did not significantly alter serum oxidative status, blood pressure or liver and kidney function compared to the control.<br>In vitro evaluation of P. palmata extracts and protein hydrolysates identified a significant induction of the inflammation marker interleukin-8 (IL-8) secretion by Caco-2 cells
Alaria esculenta	Insulin-like growth factor 1 (IGF-1) which is the marker for postmenopausal breast cancer	Teas et al 2011	Human RCT<br>N = 30 healthy postmenopausal women (mean age 58 y)	14-wk, placebo-controlled crossover clinical trial: Participants consumed 5 g/day placebo or seaweed (Alaria esculenta) in capsules for 7 wk. During the 7th wk, a high-soy protein isolate powder was added (2 mg/kg body weight aglycone equivalent isoflavones). Overnight fasting blood samples were collected after each intervention period.	Soy significantly increased serum IGF-1 concentrations compared to the placebo. The combination of seaweed and soy significantly reduced this increase<br>Concurrent seaweed and soy consumption may be important in modifying the effect of soy on IGF-1 serum concentrations.
Xanthigen (brown marine algae fucoxanthin + pomegranate seed oil (PSO)	Body weight<br>Body fat<br>Liver lipids<br>Blood biochemistry<br>Resting energy expenditure (REE) in obese, non-diabetic female volunteers with non-alcoholic fatty liver disease (NAFLD) and normal	Abidov et al 2010	Human RCT<br>N = 151 non-diabetic, obese premenopausal women	Weekly assessment of different types of analysis for 16 weeks after consuming the daily dietary intakes	Xanthigen promoted weight loss, reduced body and liver fat content, and improved liver function tests in obese non-diabetic women.<br>Xanthigen and Fucoxanthin increased REE.

	liver fat (NLF) content.				
Alaria esculenta	Urinary estrogen metabolite analysis<br>Urinary phytoestrogen analysis<br>Serum sex hormone analyses	Teas et al 2009	Human RCT<br>N = 15 healthy postmenopausal women	7 wk of consumption either 5 g/d seaweed (Alaria) or placebo (maltodextrin). During wk 7, participants also consumed a daily soy protein isolate (2 mg isoflavones/kg body weight). After a 3-wk washout period, participants were crossed over to the alternate supplement schedule.<br>Samples were collected a total of 6 times (blood samples and 48-h urine collection) for further analysis.	Seaweed favourably alters estrogen and phytoestrogen metabolism and these changes likely include modulation of colonic bacteria.
Alaria esculenta	Urinary I/C serum thyroxine free thyroxine index<br>Total triiodothyronine<br>Thyroid stimulating hormone (TSH)	Teas et al 2007	Human RCT<br>N = 25 healthy postmenopausal women (mean age 58 y)	Ten capsules (5 g/day) of placebo or seaweed (Alaria esculenta), providing 475 microg of iodine/day, were consumed daily for 7 weeks. A powdered soy protein isolate providing 2 mg of isoflavones/kg of body weight, was given daily during the last week of each treatment arm.<br>Compared plasma & 48-h urine samples pre and post intervention for different types of analysis	Soy supplementation did not affect thyroid end points. Seven weeks of 5 g/day seaweed supplementation was associated with a small but statistically significant increase in TSH.

Supplementary Table S1b. Algae Protein, Animal Studies

Protein type & form	Biomarker / Health measures	Reference	Study type	Study summary	Outcome
Spirulina	Liver bacterial translocation<br>Hematological profile<br>Circulating inflammatory<br>Redox markers	Mullenix et al 2021	Animal-<br>N = 180<br>One-day-old 708 male broilers	Compare standard diet as a control, low protein diet, and low protein diet supplemented with 100 g/kg of Spirulina	Spirulina reduces systemic inflammation- and bacterial translocation-induced by a low protein diet
Red seaweed Palmaria palmata protein hydrolysate	Blood pressure	Fitzgerald et al 2014	Animal-spontaneously hypertensive rats<br>In vitro digestion	Compare systolic blood pressure (SBP) of rats after 24h fed with seaweed protein hydrolysate containing the tridecapeptide IRLIIVLMPILMA and the synthetic peptide.	The seaweed protein derived hydrolysate has potential for use as antihypertensive agents<br>The tridecapeptide is cleaved and activated to the dipeptide IR when it travels through the GI tract.
Chlorella protothecoides<br>Whole Algalin Protein (WAP)	Body weight<br>Food consumption<br>Ophthalmology<br>Urinalysis<br>Hematology<br>Clinical chemistry<br>Gross pathology<br>Organ weights<br>Histopathology	Szabo et al 2013	Animal-13-week feeding trial in rodents<br>In vitro	Compare 13-week feeding trial with 0, 25,000, 50,000 or 100,000 ppm WAP for 92–93 days	No mutagenicity occurred in Salmonella typhimurium or Escherichia coli tester strains with or without mutagenic activation.<br>No clastogenic response occurred in bone marrow from mice administered a single oral dose (2000 mg/kg WAP).<br>Skin sensitization was not induced by WAP via HRIPT, indicating little potential for food allergy.

Supplementary Table S2. Cereal Protein, Human Studies

Protein type & form	Biomarker /Health measures	Reference	Study type	Study summary	Outcome
Wheat protein<br>Hydrolysate (WPH: 2 g) and/or l-arginine (ARG: 3.2 g)	Satiating effects<br>Plasma serotonin<br>Gastric emptying	Stoeger et al 2019	Oral glucose tolerance test (oGTT)-based bolus interventions of 75 g glucose + 0.15 mg nonivamide (NV control) are tested with/without combination of a wheat protein hydrolysate (WPH: 2 g) and/or l-arginine (ARG: 3.2 g) for their satiating effects in 27 moderately overweight male subjects.	Compared to NV control intervention, ARG and WPH + ARG treatment both reduce ($p < 0.01$) total calorie intake from a standardized breakfast by -5.9 ± 4.15% and -6.07 ± 4.38%, respectively. For the WPH + ARG intervention, increased mean plasma serotonin concentrations (AUC: 350 ± 218), quantitated by ELISA, and delayed gastric emptying, assessed by (13) C-Na-acetate breath test (-2.10 ± 0.51%, $p < 0.05$), are demonstrated compared to NV control. Correlation analysis between plasma serotonin and gastric emptying reveals a significant association after WPH ± ARG intervention ($r = -0.396$, $p = 0.045$).	Combination of WPH and ARG enhances the satiating effect of nonivamide, providing opportunities to optimize satiating food formulations by low amounts of the individual food constituents.
Wheat protein<br>35-g bolus of wheat protein hydrolysate compared with casein and whey protein.	Assessing postprandial plasma amino acid concentrations and muscle protein synthesis rates	Gorissen et al. 2016	Sixty healthy older men [mean ± SEM age: 71 ± 1 y; body mass index (in kg/m(2)): 25.3 ± 0.3] received a primed continuous infusion of l-[ring-(13)C6]-phenylalanine and ingested 35 g wheat protein (n = 12), 35 g wheat protein hydrolysate (WPH-35; n = 12), 35 g micellar casein (MCas-35; n = 12), 35 g whey protein (Whey-35; n = 12), or 60 g wheat protein hydrolysate (WPH-60; n = 12).	The postprandial increase in plasma essential amino acid concentrations was greater after ingesting Whey-35 (2.23 ± 0.07 mM) than after MCas-35 (1.53 ± 0.08 mM) and WPH-35 (1.50 ± 0.04 mM) ($P < 0.01$). Myofibrillar protein synthesis rates increased after ingesting MCas-35 ($P < 0.01$) and were higher after ingesting MCas-35 (0.050% ± 0.005%/h) than after WPH-35 (0.032% ± 0.004%/h) ($P = 0.03$). The postprandial increase in plasma leucine concentrations was greater after ingesting Whey-35 than after WPH-60 (peak value: 580 ± 18 compared with 378 ± 10 μM, respectively; $P < 0.01$), despite similar leucine contents (4.4 g leucine). Nevertheless, the ingestion of WPH-60 increased myofibrillar protein synthesis rates above basal rates (0.049% ± 0.007%/h; $P = 0.02$).	The myofibrillar protein synthetic response to the ingestion of 35 g casein is greater than after an equal amount of wheat protein. Ingesting a larger amount of wheat protein (i.e., 60 g) substantially increases myofibrillar protein synthesis rates in healthy older men
Wheat protein<br>(gluten)<br>& Rice protein<br>the effect of four protein hydrolysates from vegetable (pea,	Glucagon and insulin responses	Claessens et al 2009	Eight healthy normal-weight male subjects participated in this study. The study employed a repeated-measures design with Latin square randomization and single-blind trials.	All protein hydrolysates induced an enhanced insulin secretion compared to maltodextrin alone and a correspondingly low plasma glucose response. A significant difference was observed in area under the curve (AUC) for plasma glucagon between protein hydrolysates and the maltodextrin control drink ($P<0.05$). Gluten protein hydrolysate induced the lowest glucagon response.	High amino-acid-induced glucagon response does not necessarily go together with low insulin response. Protein hydrolysate source affects AUC for glucagon more profoundly than for insulin, although the protein load used in this study seemed to be at lower level for significant physiological effects.

gluten, rice and soy) and two protein hydrolysates from animal origin (whey and egg)					
Wheat protein<br>starch in the control bread was replaced by protein (wheat gluten)	Urinary calcium losses and bone turnover	Jenkins et al 2003	Randomized controlled cross-over study. SETTING: Teaching hospital and university. SUBJECTS: Twenty hyperlipidemic men and postmenopausal women (age 56+/-2 y) completed the study. INTERVENTION: One-month test and control phases during which subjects consumed equi-energy metabolic diets high in calcium (1578 and 1593 mg/day, respectively). On the test diet 11% of total dietary energy from starch in the control bread was replaced by protein (wheat gluten), resulting in 27% of energy from protein on the test diet vs 16% on the control diet.	Compared with the control diet, at week 4, the test diet increased mean (+/-s.e.m.) 24 h urinary output of calcium (139+/-15 vs 227+/-21 mg, P=0.004). The treatment difference in urinary calcium loss correlated with the serum anion gap as a marker of metabolic acid production (r=0.57, P=0.011). Serum calcium levels were marginally lower 2.41+/-0.02 vs 2.38+/-0.02 mmol/l (P=0.075), but there was no significant treatment difference in calcium balance, possibly related to the high background calcium intake on both diets.	In the presence of high dietary calcium intakes the vegetable protein gluten does not appear to have a negative effect on calcium balance despite increased urinary calcium loss.
Wheat protein<br>Gluten	Metabolic effects<br>Renal function	Jenkins et al 2001	Twenty hyperlipidemic men and women consumed isoenergetic test (high-protein) and control metabolic diets for 1 mo in a randomized crossover design. In the high-protein diet, 11% of the total dietary energy from starch in the control bread was replaced by vegetable protein (wheat gluten), resulting in 27% of total energy from protein compared with 16% in the control diet.	Compared with the control, the high-protein diet resulted in lower serum concentrations of triacylglycerol (by 19.2 +/- 5.6%; P = 0.003), uric acid (by 12.7 +/- 2.0%; P < 0.001), and creatinine (by 2.5 +/- 1.1%; P = 0.035) and higher serum concentrations of urea (by 42.2 +/- 5.8%; P < 0.001) and a higher 24-h urinary urea output (by 99.2 +/- 17.2%; P < 0.001). No significant differences were detected in total or HDL cholesterol or in the renal clearance of creatinine. LDL oxidation, assessed as the ratio of conjugated dienes to LDL cholesterol in the LDL fraction, was lower with the high-protein diet (by 10.6 +/- 3.6%; P = 0.009)	High intakes of vegetable protein from gluten may have beneficial effects on cardiovascular disease risk by reducing oxidized LDL, serum triacylglycerol, and uric acid. Further studies are required to assess the longer-term effects on renal function.
Wheat protein<br>0.4 g hydrolysate plus free leucine and	Postexercise muscle glycogen synthesis	van Loon, Saris et al, 2000	Eight trained cyclists visited the laboratory 3 times, during which a control beverage and 2 other beverages were tested. After the subjects participated in a strict glycogen-depletion protocol, muscle	Plasma insulin responses in the Carb + Pro and Carb + Carb trials were higher than those in the Carb trial (88 +/- 17% and 46 +/- 18%; P < 0.05). Muscle glycogen synthesis was higher in both trials than in the Carb trial (35. 4 +/- 5.1 and 44.8 +/- 6.8 compared with 16.6 +/- 7.8 micromol glycosol units*g dry wt(-)(1)*h(-)(1), respectively; P < 0.05)	Addition of a mixture of protein hydrolysate and amino acids to a carbohydrate-containing solution (at an intake of 0.8 g carbohydrate*kg(-)(1)*h(-)(1)) can stimulate glycogen synthesis. However,

phenylalanine*kg(-)(1)*h(-)(1)<br><br>ingestion of carbohydrate and wheat protein hydrolysate with and without free leucine and phenylalanine			biopsy samples were collected. The subjects received a beverage every 30 min to ensure ingestion of 0.8 g carbohydrate*kg(-)(1)*h(-)(1) (Carb trial), 0.8 g carbohydrate*kg(-)(1)*h(-)(1) plus 0.4 g wheat protein hydrolysate plus free leucine and phenylalanine*kg(-)(1)*h(-)(1) (proven to be highly insulinotropic; Carb + Pro trial), or 1.2 g carbohydrate*kg(-)(1)*h(-)(1) (Carb + Carb trial). After 5 h, a second biopsy was taken		glycogen synthesis can also be accelerated by increasing carbohydrate intake (0.4 g*kg(-)(1)*h(-)(1)) when supplements are provided at 30-min intervals.
Wheat protein<br>ingestion of carbohydrate and wheat protein hydrolysate with and without free leucine and phenylalanine	Postexercise insulin response	van Loon, Kruijshoo et al 2000	After an overnight fast, eight male cyclists visited laboratory on five occasions, during which a control drink and two different beverage compositions in two different doses were tested. After they performed a glycogen-depletion protocol, subjects received a beverage (3.5 mL. kg(-1)) every 30 min to ensure an intake of 1.2 g. kg(-1). h(-1) carbohydrate and 0, 0.2 or 0.4 g. kg(-1). h(-1) protein hydrolysate (and amino acid) mixture.	ingestion of the beverages containing wheat protein hydrolysate, leucine and phenylalanine resulted in a marked increase in insulin response (+52 and + 107% for the 0.2 and 0.4 g. kg(-1). h(-1) mixtures, respectively; P: < 0. 05) compared with the carbohydrate-only trial). A dose-related effect existed because doubling the dose (0.2-0.4 g. kg(-1). h(-1)) led to an additional rise in insulin response (P: < 0.05). Plasma leucine, phenylalanine and tyrosine concentrations showed strong correlations with the insulin response (P: < 0.0001).	This study provides a practical tool to markedly elevate insulin levels and plasma amino acid availability through dietary manipulation, which may be of great value in clinical nutrition, (recovery) sports drinks and metabolic research.
Oat Protein<br>& Rice protein<br>Beverage supplemeneted with added with 24 g oat, pea or rice proteins	Postprandial glucose and insulin response Insulin, triglycerides, glucose-dependent insulinotropic peptide (GIP), glycogen-like peptide-1 (GLP-1) and	Tan et al 2018	a randomized, crossover acute feeding study consisting of four treatments: chocolate beverage alone (50 g carbohydrate), or added with 24 g oat, pea or rice proteins. Twenty Chinese males (mean ± SD age 26 ± 5 years; body mass index 21.5 ± 1.7 kg/m(2)) ingested the test drink after an overnight fast.	Significant interaction effects were found in postprandial glucose excursions (time × protein effects, p = 0.003). Glucose iAUC was lower in pea and rice proteins, although not significantly (p > 0.385). Insulin iAUC was significantly higher in the oat (p = 0.035) and pea (p = 0.036) protein beverages. GIP and GLP-1 release in a sub-sample (n = 10) followed a comparable order as insulin release (p = 0.397 and 0.454, respectively). Significant interaction effects were found in fullness ratings (p = 0.024), and a trend of greater suppression of hunger and desire-to-eat was also documented (p = 0.088 and 0.080, respectively)	Plant proteins altered the glycemic and appetitive responses of Asian males to a sugar-sweetened beverage. Food-based interventions are useful in promoting glycemic control

	appetite responses				
Rice protein<br>Drink supplement -- Participants consumed 40 g of either whey hydrolysate (WH, n = 9), whey isolate (WI, n = 6), rice and pea combination (RP, n = 6), or placebo (PL, n = 6)	Muscle recovery	Saracino et al 2020	Twenty-seven recreationally active, middle-aged men performed 5 sets of 15 repetitions of maximal eccentric voluntary contractions (ECC) for the knee extensors (ext) and flexors (flex), respectively, in the morning. Participants consumed 40 g of either whey hydrolysate (WH, n = 9), whey isolate (WI, n = 6), rice and pea combination (RP, n = 6), or placebo (PL, n = 6) 30 min pre-sleep on the day of ECC and the following two nights.	Plasma creatine kinase (CK), interleukin-6 (IL-6), and interleukin-10 (IL-10) were measured at pre, immediately post (+0), +4, +6, +24, +48, and +72 h post-ECC. Isometric (ISOM) and isokinetic (ISOK) maximal voluntary contraction force were measured at pre, immediately post (+0), +24, +48, and +72 h post-ECC. Muscle soreness, thigh circumference, and HOMA-IR were measured at pre, +24, +48, and +72 h post-ECC. CK was increased at +4 h post-ECC, remained elevated at all time points compared to baseline ($p < 0.001$), and was significantly greater at +72 h compared to all other time points ($p < 0.001$). IL-6 was increased at +6 h ($p = 0.002$) with no other time differing from baseline. ISOMext was reduced after ECC ($p = 0.001$) and remained reduced until returning to baseline at +72 h. ISOMflex, ISOKext, and ISOKflex were reduced after ECC and remained reduced at +72 h ($p < 0.001$). Muscle soreness increased post-ECC ($p < 0.001$) and did not return to baseline. Thigh circumference ($p = 0.456$) and HOMA-IR ($p = 0.396$) did not change post-ECC. There were no significant differences between groups for any outcome measure.	These data suggest that middle-aged men consuming 1.08 ± 0.02 g/kg/day PRO did not recover from damaging eccentric exercise at +72 h and that pre-sleep protein ingestion, regardless of protein source, did not aid in muscle recovery when damaging eccentric exercise was performed in the morning.<br><br>The primary findings of the present study were that whey and plant-based protein consumed pre-sleep failed to consistently improve muscle function during the 72 h recovery period and whey and plant-based protein consumed pre-sleep failed to reduce blood markers of muscle damage and inflammation or muscle recovery
Rice protein<br>The study objectives were to determine the bioavailability of methionine in rice and chickpeas separately and to assess the effect of complementation of chickpeas and rice	Bioavailability using indicator amino acid oxidation (IAAO) method, with l-[1-13C] phenylalanine as the indicator	Rafii et al 2020	Eleven healthy young men (<30 y, BMI <25 kg/m2) were studied in a repeated-measures design	The bioavailability of methionine from rice and from chickpeas was 100% and 63%, respectively. Complementation of cooked chickpeas with rice decreased the oxidation of l-[1-13C] phenylalanine by up to 14% ($P < 0.05$), suggesting an improved protein quality of the combined chickpeas plus rice protein.	When chickpeas are the main protein source in the diet of young adult men, the combination of rice and chickpeas in a 3:1 ratio is recommended to improve dietary protein quality.
Rice protein<br>hydrolysate	Modulation of inflammation markers.	Rein et al 2019	10 healthy subjects consumed the peptides with a single bolus of 20 g protein hydrolysate	The 24-hour kinetic study revealed a slight suppression of pro-inflammatory TNF-α, IP-10 and NOx, whereas IL-6 increased temporarily (timepoints 2-12 hours). These markers returned to the baseline after 24 hours whereas	Consumption of a single dose protein hydrolysate containing immune modulatory peptides induced a mild

				others were not affected significantly (IL-10, hs-CRP, IL-8, and MCP-1).	temporary response most likely through intestinal signaling.
Rice protein isolate	Body composition, psychometric scores of perceived recovery, soreness, or readiness to train	Joy et al 2013	24 college-aged, resistance trained males were recruited for this study. Subjects were randomly and equally divided into two groups, either consuming 48 g of rice or whey protein isolate (isocaloric and isonitrogenous) on training days. Subjects trained 3 days per week for 8 weeks as a part of a daily undulating periodized resistance-training program. The rice and whey protein supplements were consumed immediately following exercise.	No detectable differences were present in psychometric scores of perceived recovery, soreness, or readiness to train ($p > 0.05$). Significant time effects were observed in which lean body mass, muscle mass, strength and power all increased and fat mass decreased; however, no condition by time interactions were observed ($p > 0.05$).	Both whey and rice protein isolate administration post resistance exercise improved indices of body composition and exercise performance; however, there were no differences between the two groups.
Rice Protein concentrate consume 24-g doses of rice (n =12, Growing Naturals, LLC) or whey (n=12, NutraBio Labs, Inc.) protein concentrate for 8 weeks while completing a standardized resistance training program	Body composition, muscle strength, endurance and anaerobic capacity	Moon et al 2020	Healthy resistance-trained males (n =24, 32.86.7years, 179.38.5cm, 87.4 +/- 8.5kg, 27.2 +/- 1.9kg/m(2), 27.8 +/- 6.0% fat) were randomly assigned and matched according to fat-free mass to consume 24-g doses of rice (n =12, Growing Naturals, LLC) or whey (n=12, NutraBio Labs, Inc.) protein concentrate for 8 weeks while completing a standardized resistance training program.	No changes ($p > 0.05$) in dietary status occurred within or between groups (34 +/- 4kcal/kg/day, 3.7 +/- 0.77g/kg/day, 1.31 +/- 0.28g/kg/day, 1.87 +/- 0.23g/kg/day) throughout the study for daily relative energy (34 +/- 4 kcals/kg/day), carbohydrate (3.7 +/- 0.77g/kg/day), fat (1.31 +/- 0.28g/kg/day), and protein (1.87 +/- 0.23g/kg/day) intake. Significant main effects for time were revealed for body mass ($p = 0.02$), total body water ($p = 0.01$), lean mass ($p = 0.008$), fat-free mass ($p = 0.007$), BP 1RM ($p = 0.02$), BP volume ($p = 0.04$), and LP 1RM ($p = 0.01$). Changes between groups were similar for body mass (-0.88, 2.03kg, $p = 0.42$), fat-free mass (-0.68, 1.99kg, $p = 0.32$), lean mass (-0.73, 1.91kg, $p = 0.37$), fat mass (-0.48, 1.02kg, $p = 0.46$), and % fat (-0.63, 0.71%, $p = 0.90$). No significant between group differences were seen for BP 1RM (-13.8, 7.1kg, $p = 0.51$), LP 1RM (-38.8, 49.6kg, $p = 0.80$), BP RTF (-2.02, 0.35 reps, $p = 0.16$), LP RTF (-1.7, 3.3 reps, $p = 0.50$), and Wingate peak power (-72.5, 53.4 watts, $p = 0.76$) following the eight-week supplementation period	Eight weeks of daily isonitrogenous 24-g doses of rice or whey protein in combination with an eight-week resistance training program led to similar changes in body composition and performance outcomes
Buckwheat or corn protein instant porridges were developed: BW -	Lipid profile and inflammation	Misan et al 2017	A randomised, cross-over intervention study was performed among 34 participants with mild to moderate hypercholesterolemia.	Intake of BW porridge significantly reduced serum levels of total cholesterol, LDL cholesterol, triacylglycerol and, uric acid and, significantly increased serum adiponectin levels, HDL cholesterol, and fat-free mass.	Intake of BW porridge as a functional food may improve lipid profile and may reduce inflammation

enriched high protein (A), corn-based high-protein (B) and corn-based non-protein (C)					
Barley protein	Serum lipids	Jenkins et al 2010	Twenty-three hypercholesterolemic men and postmenopausal women completed a randomized crossover study comparing a bread enriched with either barley protein or calcium caseinate [30 g protein, 8374 kJ (2000 kcal)] taken separately as two 1-mo treatment phases with a minimum 2-wk washout.	Palatability, satiety, and compliance were similar for both the barley protein- and casein-enriched breads, with no differences between the treatments in effects on serum LDL cholesterol or C-reactive protein, measures of oxidative stress, or blood pressure.	no adverse effects were observed on cardiovascular risk factors, barley protein remains an additional option for raising the protein content of the diet.
Rye protein<br>whole-grain rye porridge enhanced by replacing part of the rye with fermented dietary fibre and plant protein	Appetite, breath hydrogen and methane, glucose, insulin and glucagon-like peptide-1 (GLP-1) responses	Lee et al 2016	A randomised, cross-over study using two rye porridges (40 and 55 g), three 40-g rye porridges with addition of inulin:gluten (9:3; 6:6; 3:9 g) and a refined wheat bread control (55 g), served as part of complete breakfasts. A standardised lunch and an ad libitum dinner were served 4 and 8 h later, respectively. Twenty-one healthy men and women, aged 23-60 years, with BMI of 21-33 kg/m2 participated in this study	Before lunch, the 55-g rye porridges lowered hunger by 20 % and desire to eat by 22 % and increased fullness by 29 % compared with wheat bread ($P<0{\cdot}05$). Breath hydrogen increased proportionally to dietary fibre content ($P<0{\cdot}05$). Plasma glucose after lunch was 6 % lower after the 55-g rye porridges compared with wheat bread ($P<0{\cdot}05$) and correlated to breath hydrogen ($P<0{\cdot}001$). No differences were observed in ad libitum food intake, insulin or GLP-1.	no further increase in satiety was observed when replacing part of the rye with inulin and gluten compared with plain rye porridges.
Amaranth seed protein concentrate	Lipid content in serum and liver tissue;<br>oxidative stress in liver	Escudero et al 2006	Animal - rat	Control diet = casein; experimental diet = amaranth protein concentrate	No difference in TCs; decrease in TGs; fatty acid synthase decreased; antioxidant protection.<br>Hypotriglyceridemic effect and antioxidant protection of amaranth protein compared with casein.

Supplementary Table S3. Fresh Fruits and Vegetable Protein, Human Studies

Protein type & form	Biomarker / Health measures	Reference	Study type	Study summary	Outcome
Potato and rice protein isolates	Glycaemic indices and gut hormones	Lorinczova et al 2021	Single-blind, triple-crossover design study with nine male participants (30.8 +/- 9.3 yrs)	The acute effects on glycaemic indices, gut hormones, and subjective appetite ratings of two high-quality, plant-derived protein isolates (potato and rice)	Study suggests that both plant-derived proteins had a lower insulinaemic response and improved glucose maintenance compared to whey protein
Potato Protein Isolate	Skeletal muscle myofibrillar protein synthesis (MPS)	Oikawa et al 2020	In a single blind, parallel-group design, 24 young women (21 ± 3 years, n = 12/group) consumed a weight-maintaining baseline diet containing 0.8 g/kg/d of protein before being randomized to consume either 25 g of PP twice daily (1.6 g/kg/d total protein) or a control diet (CON) (0.8 g/kg/d total protein) for 2 weeks	Ingestion of PP stimulated MPS by 0.14 ± 0.09 %/d at Rest, and by 0.32 ± 0.14 %/d in the Exercise limb. MPS was significantly elevated by 0.20 ± 0.11 %/d in the Exercise limb in CON (P = 0.008). Consuming PP to increase protein intake to levels twice the recommended dietary allowance for protein augmented rates of MPS.	Potato protein is a high-quality, plant-based protein supplement that augments myofibrillar protein synthesis MPS at rest and following resistance exercise in healthy young women.
Oat, pea and potato protein blend	Appetite rating, insulin response and metabolic measures	Dougkas et al 2018	28 males consumed three isoenergetic (1674 kJ) rice puddings matched for energy density and macronutrient content as breakfast (25% E from protein) in a single-blind, randomised, cross over design. Groups:<br>animal proteins [milk (AP)],<br>a blend of plant proteins [oat, pea and potato (VP)]<br>50:50 mixture of the two (MP)<br>carbohydrate-rich meal (CHO)	No differences in subjective appetite ratings were observed after consumption of the AP, VP and MP.	Manipulating the protein source of foods consumed as breakfast, elicited comparable effects on appetite and EI at both laboratory and free-living environment in healthy men.
Potato isolates: a high (HMW) and a low (LMW) molecular weight fraction	Digestion kinetics	He et al 2013	Human study: Ingestion of 20 g of proteins by eight healthy subjects (following a randomized, double-blind, cross-over design).<br>In vitro studies	Contrary to whey and casein, HMW and LMW did not result in any changes in plasma insulin or glucose levels.	This study provides insights in digestion of native potato protein isolates to assist their use as protein sources in food applications.

Supplementary Table S4. Insect and Snail Protein, Human, Animal and In Vitro Studies

Protein type & form	Biomarker / Health measures	Reference	Study type	Study summary	Outcome
Cricket Protein hydrolysates	Antihypertensive<br>Anti-glycemic<br>Anti-inflammatory peptides	Hall et al 2020	In vitro gastrointestinal digests	Identify and characterize the bioactive peptides responsible for the ACE and DPP-IV-inhibiting activity, and to evaluate their anti-inflammatory activity on RAW 264.7 macrophage cells.	• Cricket protein hydrolysates contain potent peptides with potential ACE, alpha-glucosidase, and alpha-amylase inhibiting capacity<br>• Cricket peptides, alone or in the form of functional foods, might help for conditions associated with inflammation and hypertension.<br>• More research needed, using in vivo models and clinical trials.
Cricket (Gryllodes sigillatus)	Malnutrition recovery<br>Inflammation and metabolism biomarkers	Bergmans et al 2020	In vivo, rat<br>Mice<br>N=65	Study to test if cricket protein can help in the recovery of malnutrition, comparing it to peanut and milk-based diets	• Cricket protein diet performed equally well as peanut and milk diets, but there were differences in immune and metabolic markers that require further investigation.
Cricket<br>Termite<br>Moth larvae<br>Grasshopper	Health measures	Inje et al 2018	In vivo, rat<br>N = 36<br>weanling albino<br>28 days	6 groups: 5 isonitrogenous & isocaloric insect or casein diets (10% protein)<br>+ a nitrogen-free diet control.	• Cricket was found to have the highest amino acid score (0.91), protein efficiency ratio (1.78), net protein ratio (3.04) biological value (93.02%) and protein<br>• organ body weight ratios of the liver, spleen, lung and heart of rats placed on the insect supplemented diets were not significantly different ($p > 0.05$) from those fed with casein and basal diets digestibility corrected for amino acid score (0.73)<br>• Serum total protein concentrations in rats fed with cricket diet were not significantly different ($p > 0.05$) from those fed casein diet<br>• serum LDL cholesterol concentration was lowest in rats fed cricket diet<br>• Concluded insects are safe & nutritious
Cricket (Gryllodes sigillatus)	Microbiota	Stull et al 2018	Humans<br>N=20	Evaluates the effects of consuming 25 grams/day whole cricket powder on gut microbiota composition, while assessing safety and tolerability	• Cricket consumption is tolerable and non-toxic at the studied dose.<br>• Cricket powder supported growth of the probiotic bacterium, Bifidobacterium animalis (increased 5.7-fold).<br>• Cricket consumption was associated with reduced plasma TNF-alpha.<br>• Authors suggest that eating crickets may improve gut health and reduce systemic inflammation; but, more research is needed.

Cricket (Gryllodes sigillatus Insect protein hydrolysates)	Antioxidant angiotensin converting enzyme (ACE) dipeptidyl peptidase-4 (DPPIV)-inhibition of the cricket protein hydrolysates (CPH)	Hall et al 2018	In vitro (simulated gastrointestinal digestion, SGD)	To evaluate the effect of enzymatic hydrolysis on bioactive properties of cricket protein hydrolysates before and after SGD.	• Hydrolysates displayed good ACE-, DPP-IV inhibition, and antioxidant activity.<br>• Bioactivity improved after SGD.
Crickets (Gryllodes sigillatus) Locusts (Schistocerca gregaria)	Antioxidant and anti-inflammatory properties Identification of bioactives	Zielinska et al 2017	In vitro	To determine the effect of heat treatment of edible insects on antioxidant and anti-inflammatory activities of peptide fractions from hydrolysates obtained by in vitro gastrointestinal digestion.	• Heat treatment had positive effect on antioxidant and anti-inflammatory properties.<br>• Cricket showed highest anti-radical (LOX and COX-2 inhibitory) activity.<br>• Peptide fraction from mealworm protein showed highest Fe2+ chelating ability.
Mealworm (Yellow) Tenebrio molitor larva (fermentation extract, TMP)	Weight & fat mass Glucose tolerance & insulin resistance (IR) Proteomics Hepatic steatosis	Ham et al 2021	In vivo – obese mice, induced by high fat diet 12 week intervention 4 diets n=10 per group	Compared health effects of protein from soy (SP) or TMP, in low or high-fat (HF) diets. Note previous studies have shown positive effect of mealworm diet on NAFLD and hepatic steatosis. Defatted and freeze-dried fermented MW extract (TMP) has higher free amino acids and EAAs than non-fermented.	• TMP reduced body weight, weight gain, fat mass, fat size, glucose tolerance and IR compared to HF-SP diet.<br>• TMP alleviated hepatic steatosis (lipid contents & droplets) in high fat diet groups & down regulated associated genes.<br>• TMP sig downregulated proteins involved in glucose, lipid & amino acid metabolism, oxid and ER stress.<br>• TMP helped improve high-fat diet-induced obesity, steatosis, IR in mice.
Mealworm (lesser) (Alphitobius diaperinus)	Food intake regulation	Miguens-Gomez Et al 2020	- ex vivo models (human, N=10 and pig intestine, N=3) with in vitro digestions<br>- in vivo in rats (N=10)	Comparison of the interaction of insect, beef and almond protein with the gastrointestinal tract.	• Insect and beef are most effective reducing CCK<br>• GLP-1 levels were increased with insect protein<br>• Insect protein reduced ghrelin secretion in human and pig<br>• Insect protein increased food intake of rats
Mealworm (yellow, Tenebrio molitor) Cricket and Silkworm proteins	Anti-inflammatory activity	Yoon et al 2019	In vitro Insect protein hydrolysates	Examined effect of different hydrolysis protocols with commercial enzymes. Anti-inflammatory activity assessed via nitric oxide production from macrophages.	• Processing changed the anti-inflammatory activity of the proteins, potentially reducing their effectiveness.
Mealworm (Yellow) Tenebrio molitor	Metabolic health	Gessner et al 2019	In vivo Lean & obese Zucker rats	Control diet: 20% Casein<br>50 or 100% isonitrogenous replacement with insect meal (MW).	• Significant reduction in transcription pf genes involved in fatty acid, triacylglycerol (TG) and cholesterol biosynthesis in liver of rats fed MW.

(added from Review van Huis 2021)			N = 12/group		• Liver lipid enzymes were significantly reduced, as were liver and plasma TG concentrations.<br>• Plasma and liver homocysteine reduced by 20-30% in MW fed animals.<br>• MW meal had pronounced lipid lowering effects, and may be useful in human health.
Mealworm (lesser, Alphitobius diaperinus) Protein isolate and protein concentrate)	Bioactive for Type 2 diabetes<br>Presence and activity of DPP-IV inhibitors	Lacroix et al 2019	In vitro	Dipeptidyl-peptidase IV (DPP-IV) involved in inactivation of incretins, gut-derived hormones, important in glycemic regulation.<br>Inhibition of DPP-IV enzyme is a strategy for T2 diabetes. 10 dietary protein-derived "gliptins" already approved as therapeutics. Can DPP-IV inhibitors can be generated by digestion or enzymatic hydrolysis of lesser mealworm protein isolate and concentrate ?	• DPP-IV inhibitors can be generated from lesser mealworm protein<br>• insects may serve as functional food ingredients
Mealworm	Antioxidant and anti-inflammatory properties<br>Identification of bioactives	Zielinska et al 2018	In vitro	To determine the effect of heat treatment of edible insects on antioxidant and anti-inflammatory activities of peptide fractions from hydrolysates obtained by in vitro gastrointestinal digestion.	• Heat treatment had positive effect on antioxidant and anti-inflammatory properties.<br>• Peptide fraction from mealworm protein showed highest Fe2+ chelating ability.
Mealworm (lesser, Alphitobius diaperinus) (protein isolate)	Post-prandial amino acid (AA) avail<br>AA profile in blood	Vangsoe et al 2018	Human RCT<br>N = 6 males<br>Crossover design	4 test supplements over 4 days, containing protein isolate from lesser mealworm, whey isolate, and soy isolate, or water (control).	• A significant rise in blood concentration of essential amino acids (EAA), branched-chain amino acids (BCAA) and leucine was detected over the 120 min period for all protein supplements<br>• AA profile was significantly greater after whey than soy and insect protein ($p < 0.05$).<br>• Insect protein was highest at 120 minutes, suggesting slower digestion.<br>• Conclusion: all 3 protein isolates increased EAA, BCAA and Leu over 120 min.
Mealworm (Yellow) Tenebrio molitor	Metabolism<br>Metabolic amine profiles in serum & urine	Kar et al 2017(a)	In vivo, mice<br>28 days	Compared diets of yellow mealworm, soybean meal, casein, partially de-lactosed whey powder, spray-dried plasma protein, and wheat gluten meal.	• Serum is recommended over urine to screen for the amine metabolic endophenotype.<br>• Metabolites such as alpha-aminobutyric acid and 1-methylhistidine are sensitive indicators of too much or too little availability of specific amino acids in the different protein diets.<br>• Amine metabolic profiles can be useful for assessing the nutritional quality of different protein sources.

Mealworm (Yellow)<br>Tenebrio molitor<br>Powdered, Freeze dried, larvae (TML)<br>Added from review Mishyna 2021	Lipid metabolism	Seo et al 2017	In vivo & in vitro<br>Male Balb-c mice	5 conditions including high fat diet (HFD) supplemented with 100 or 3000 mg /kg TML.<br>6 weeks duration.	• TML in vitro induced phosphorylation of adenosine monophosphate (AMP)-activated protein kinase and mitogen-activated protein kinases (MAPK).<br>• Daily oral administration of TML to obese mice fed high-fat diet attenuated body weight gain, reduced hepatic steatosis, aspartate alanine transaminase enzyme.<br>• Results suggest TML has antiobesity effect when as a food supplement.
Silkworm larvae protein isolate (SLPI)<br>Added from review Lange and Nakamura 2021	Angiotensin-converting enzyme (ACE)	Wu et al 2011	In vitro	SLPI from the fifth larval instar of the silkworm was extracted. The amino acid compositions as well as thermal properties of SLPI were evaluated. In addition, the in vitro ACE-inhibitory, antioxidant and free radical-scavenging activities of the hydrolysate obtained from SLPI using gastrointestinal proteases was investigated in detail.	• SLPI hydrolysate prepared with gastrointestinal enzymes exhibited strong in vitro ACE-inhibitory activity (IC50=8.3μg/ml) and a relatively higher DPPH radical scavenging activity (IC50=57.91 μg/ml), reducing power and ferrous ions chelating capacity (IC50=2.03 mg/ml).<br>• SLPI can be considered as a promising protein resource for preparation of the bio-peptides with ACE-inhibitory and antioxidant activities.
Silkworm<br>Freeze dried<br>Mature SW larvae powder (SMSP)<br><br>Added from Mishyna 2021	Biomarkers of alcohol-induced fatty liver disease	Lee D-Y et al 2020	In vivo<br>Male Sprague-Dawley Rats	4 weeks: AIN diet (control) or supplemented with 50mg/kg SMSP.<br>Administered 25% ethanol by oral gavage for 4 weeks.	• Ethanol-treated group on SMSP diet had significantly lower hepatic fat, modulated lipogenesis and fatty acid oxidation-related factors (sirtuin 1, AMP-activated protein kinase, and acetyl-CoA carboxylase 1).<br>• SMSP diminished the levels of triglyceride in liver tissues by as much as 35%, and decreased pro-inflammatory TNF-alpha, IL1-beta and CYT P450 2E1 generating oxidative stress.<br>• Results suggest SMSP may prevent alcoholic liver disease.
Snail<br>Meat powder (SMP)	Protein measures	Agengo et al<br>2020	In vivo – rat<br>Male weanling albino rats	Sorghum wheat buns fortified with SMP and/or skimmed milk powder.<br>9 different diets, including basal. Outcomes measured:<br><br>Protein efficiency ratio (PER)<br>Food efficiency ratio (FER)<br>Net protein retention ratio (NPRR)<br>Apparent protein digestibility (APD)<br>True protein digestibility (TPD)<br>Protein digestibility corrected amino acid score (PDCAAS)<br>Digestible indispensable amino acid score (DIAAS)	• Fortification with SMP sig enhanced PER, FER, APD and TPD.<br>• PDCAAS and DIAAS increased from 45% to 78%, and 44 to 69% respectively from unfortified buns to those with 25% SMP.<br>• SMP fortification promotes growth and rehabilitation of emaciated rats and may enhance recovery in children affected by protein malnutrition.

Snail<br>Meat protein<br>(Helix pomatia (HP),<br>Cornu.aspersa maxima (CAM) and<br>Cornu.aspersum aspersum (CAA))	Osteotropic effect of snail protein<br>Body mass, skeletal density<br>Blood Osteocalcin	Radzki et al<br>2018	In vivo, rat<br>N = 40 Wistar males<br>28 days	Compared 3 diff snail meats (Helix pomatia (HP), Cornu.aspersa maxima (CAM) and Cornu.aspersum aspersum (CAA)) with casein (control).<br>Protein 10% of diets (dry weight)	• Snail diet sig decreased bone mineral content, area of total skeleton, and isolate tibia.<br>• No effect on bone mineral density<br>• Decreased resistance to mechanical load (tibia)<br>• Snail protein diet negatively influenced metabolism of bone tissue, bones smaller and weaker.
Snail<br>Foot muscle protein hydrolysates (SFMPH)<br>Achatina fulica	ACE inhibition<br>Anti-hypercholesterol-emic activity	Huang et al<br>2017	In vitro	Inhibition of angiotensin-I converting enzyme (ACE) is a target for blood pressure management therapeutics. SFMPHs were further hydrolysed with three proteases (papain, trypsin, or alcalase).	• Alcalase hydrolysate was effective in disintegrating intact cholesterol micelles.<br>• Also showed strong ACE inhibitory activity in vitro<br>• Promising functional food ingredient
Snail (melanian)<br>Semisulco-spira libertina<br>Protein Hydrolysate	Liver and kidney damage in type II diabetes	Choi et al<br>2017	In vivo<br>Diabetic Mice<br>12 weeks	Mice adapted to a 45% kcal high fat diet (HFD), administered diets of 125, 250 and 500mg/kg snail hydrolysate (MPh).<br>Measured blood glucose & insulin, serum HDL, lesions in regions of steatohepatitis, hepatocyte hypertrophy, lipid droplet deposit-related renal tubular vacuolation, hyperplasia in pancreatic islets, insulin/glucagon-producing cell ratios in pancreas, hepatic lipid peroxidation.	• All diabetic complications signif reduced, in dose-dependent manner, in treatment groups, compared to control.<br>• MPh exerts potent anti-diabetic effects, along with the amelioration of related complications in mice with type II diabetes.<br>• The overall effects of MPh at a dose of 125 mg/kg on HFD-induced diabetes and related complications were similar or more potent than those of metformin (250 mg/kg).
Termite (winged) termite<br>(Macroterme bellicosus, MB)	Maternal acceptability of MB-protein enriched foods for infants<br>Compositional analyses of MB-enriched foods	Adepoju et al 2021	Human study<br>N = 700 respondents for consumption survey (Nigeria)<br>N = 60 nursing mothers food acceptability	MB are rich in nutrients, ~30% protein, vitamins and minerals<br>Potential complementary supplement for infants in Nigeria<br>Maize and Sorghum pap, boiled rice and yam were enriched with ground MB in the ratio 4:1 (w/w), providing 20% protein from MB, total 2-6% protein.<br>Compositional analyses conducted<br>Acceptability with nursing mothers tested.	• 95.4% of respondents reported never having negative effect after MB consumption<br>• MB significantly improved nutrient and energy content of common infant foods.<br>• MB enriched foods were acceptable to nursing mothers.
Termites<br>(Macrotermes nigeriensis)<br>Milled paste	Sensory evaluation<br>Antinutrient testing<br>Mineral content	Ojinnaka et al<br>2013	Human<br>N = 20	Substitution (0% - 20%) of wheat flour with milled paste of termite in wheat cake.<br>Protein content ranged 10 – 19.5%<br>Sensory testing	• 5% termite cake preferred<br>• Mg, Ca, K and P content increased with greater % termite inclusion

		• antinutrients (tannins, phytate, saponins, oxalate) all had low values; wheatcake product will pose no threat to human consumption.

Supplementary Table S5. Mycoprotein

Protein type & form	Biomarker / Health measures	Reference	Study type	Study summary	Outcome
Mycoprotein (MP) (Whole foods + protein shake)	Myofibrillar protein synthesis (PS)	Monteyne et al 2021	Human RCT (not blinded) N = 19 healthy older adults Mean Age: 66y	3-day high protein (1.8g/kg BM/day), isoenergetic diet. Compared muscle synthesis rate in exercised vs non-exercised leg muscle for omni diet (animal protein foods + WPI shake) vs vegan (MP whole foods + MP shake).	• PS was equivalent in exercised leg for both omni (+13%) and veg (+12%) diets. • Diets were equivalent for supporting rested and exercised daily MPS rates for healthy older adults consuming high-protein diet.
Mycoprotein (MP) (Whole foods)	Insulin sensitivity Glycaemic control Plasma lipoprotein composition Metabolomics	Coelho et al 2021	Human RCT N = 20 healthy adults	7d meals with 2 protein meals from either meat/fish (control) or MP, daily. Compared fasting plasma samples & OGTT performed pre and post intervention, plus 24h continuous BG monitoring.	• No differences between groups any insulin or glycaemic measures. • No differences for metabonomics (224 targets) • MP resulted in sig reduction in circulating cholesterol-containing lipoproteins, compared to control ($p < 0.05$)
Mycoprotein (MP) (Freeze dried isolate)	Skeletal muscle protein synthesis Serum insulin Muscle gene expression profile (mRNA)	Monteyne AJ et al (a) 2020	Human Double blind RCT N = 20 resistance-trained men Age: 22 +/- 1 y	Single bolus of mycoprotein vs leucine-matched bolus of milk protein Blood and m. vastus lateralis muscle samples were collected before exercise and protein ingestion, and following a 4-h postprandial period to assess mixed muscle fractional protein synthetic rates (FSRs) and myocellular signalling	• MP stimulates resting and post-exercise muscle protein synthesis rates, and to a greater extent than milk protein • MP supports acute tissue remodelling in response to exercise.
Mycoprotein (MP) (Freeze dried isolate, shake)	Skeletal muscle protein synthesis (PS) Serum insulin Muscle gene expression profile (mRNA)	Monteyne AJ et al (b) 2020	Human Double blind RCT N = 19 resistance-trained men Age: 22 +/-1yr	Compared whether a low dose (35 g) of mycoprotein enriched with branched-chain amino acids (BCAAs) could stimulate muscle PS to the same extent as 70 g of mycoprotein in young men, post resistance exercise.	• Plasma BCAAs increased more rapidly in BCAA-enriched group • Postprandial muscle PS increased to a greater extent in MP group ($P < 0.01$), than BCAA-enriched group. •
Mycoprotein (MP) (Whole food)	Uric acid Glucose Insulin	Coelho et al 2020	Human Double blind RCT N = 10 adults Age: 21 +/-1yr	Sandwich containing either low or high nucleotide-Quorn 'chicken' slices.	• No difference between conditions in blood glucose, serum insulin, or insulin sensitivity. • The nucleotide-rich meal increased serum uric acid for ~12 hours.
Mycoprotein (MP) (Freeze dried isolate, shake)	Postprandial hyper-aminoacidemia (HAA)	Dunlop et al 2017	Human RCT Single blind N = 12 males	Compared 20g milk protein or a mass-matched bolus of 20g MP, protein-matched 40g, 60g or 80g	• MP led to slower but more sustained HI and HAA compared to protein-matched milk protein • Bioavailability equivalent

	Hyper-insulinemia (HI)			of MP on circulating amino acids, insulin and uric acid concentrations.	• MP is a bioavailable and insulinotropic protein source to stimulate muscle synthesis.
Mycoprotein (MP) (Whole food)	Appetite<br>Glucose<br>Insulin<br>Peptide tyrosine-tyrosine (PYY)<br>Glucagon-like peptide-1 (GLP-1)	Bottin J et al 2016	Human RCT<br>N = 55 overweight adults,<br>BMI 25-32<br>Age: 18-65y	Compared low (44g), medium (88g) or high (132g) MP or isoenergetic chicken meals (risotto preparations).<br>Ad libitum energy intake assessed after 3 hours.<br>Gastric emptying rate (GER), resting energy expenditure and substrate oxidation recorded.	• MP reduced energy intake by 10% ($p = 0.0009$)<br>• MP reduced insulin concentration by 8-21% ($p = 0.004$)<br>• No difference in glucose, PYY, GLP-1, GER or energy expenditure.
Mycoprotein (MP) (Whole foods)	Cholesterol	Ruxton et al 2010	Human trial<br>Not randomised or blinded<br>N = 21 adults	Compared Quorn products consumption daily for 6 weeks, with habitual diet (control group).	• Significant reduction total cholesterol in Quorn group, in those with high baseline chol.<br>• Significant reduction in total blood chol & LDL chol.<br>• Noted compliance issues in the control group.
Mycoprotein (MP) (Whole foods)	Hunger<br>Satiety	Williamson et al<br>2006	Human RCT<br>N = 42 overweight females	Compared isocaloric preloads of pasta with MP, tofu or chicken lunches on hunger, satiety and calorie consumption later in the day.	• MP and tofu preload meals at lunch both resulted in reduced food intake afterwards, without compensation consumption at dinner.<br>• MP and tofu had greater satiety than chicken

Supplementary Table S6. Nuts and Oil Seed Protein, Human, Animal and In Vitro Studies

Protein type & form	Biomarker / Health measures	Reference	Study type	Study summary	Outcome
Canola protein isolate	Postprandial insulin, glucose, lipids, urea, amino acids and satiety	Volk et al 2020	Human - randomised controlled crossover study	A test meal without additional protein or with 28 g of rapeseed protein isolate or soy protein isolate (control). Venous blood samples were collected over a 360-min period to analyze metabolites; satiety was assessed using a visual analog scale. Postprandial levels of lipids, urea, and amino acids increased following the intake of both protein isolates.	Postprandial insulin response was lower after consumption of the rapeseed protein than after intake of the soy protein ($p < 0.05$), whereas the postmeal responses of glucose, lipids, interleukin-6, minerals, and urea were comparable between the two protein isolates.<br>Rapeseed protein exerted stronger effects on postprandial satiety than the soy protein ($p < 0.05$). The postmeal metabolism following rapeseed protein intake is comparable with that of soy protein.
Canola protein isolate and hyrolysate	Postprandial plasma amino acids and nitrogen balance	Fleddermann et al 2013	Human - RCT cross-over intervention study and animal digestibility studies	28 healthy male subjects (ø 25 years) consumed 30.0 g protein (canola protein isolate--CPI, canola protein hydrolyzate--CPH or soy protein isolate--SPI) in a randomized, double-blind, cross-over study. Blood samples were regularly drawn over the 8-h postprandial period and a 24-h urine sample was collected.	In rats, true digestibility of the canola proteins determined in a separate rat assay showed 93.3% for CPI and 97.3% for CPH<br>In humans, consumption of either 30.0 g canola protein or soy protein mixed in a drink led to significant increases in plasma amino acids after 62.3 and 83.6 min, respectively.<br>The CPH produced an earlier response compared to CPI and SPI, total amino acid response (AUC for 8 h) was comparable between all interventions<br>The nitrogen balance between the three proteins tested showed no statistical differences.
Canola protein hydrolysates	Renin inhibition Blood pressure	Alashi et al 2014	In vitro and in vivo -rats	Various hydrolysates (enzyme hydrolysis using Alcalase, chymotrypsin, pepsin, trypsin and pancreatin) tested in vitro for renin inhibition and then fed to rats	Trypsin hydrolysate was ineffective at reducing hypertension in spontaneously hypertensive rats after oral administration (200 mg/kg body weight). However, Alcalase and pepsin hydrolysates showed appreciable antihypertensive effects, with Alcalase hydrolysate producing the greatest (-34 mm Hg) and fastest (4 h) decrease in systolic blood pressure (SBP). CPI had the most prolonged (24 h) SBP-reducing effect, which is attributable to the extensive protein hydrolysis in the GIT. We conclude that the Alcalase and pepsin hydrolysates may serve as useful ingredients to formulate antihypertensive functional foods and nutraceuticals.
Canola protein	Digestibility and postprandial protein utilization	Bos et al 2007	Human feeding trial	12 human subjects testing the bioavailability and metabolic utilization of rapeseed in vivo	Postprandial biological value was high at 83.8 6 4.6%.<br>rapeseed has a low digestibility in humans compared with other plant proteins but also exhibits a very low deamination rate. Biological value of rapeseed is as high as that of milk protein.
Hemp seed protein	Blood pressure 24 hr SBP, pulse wave velocity,	Samsamikor et al 2020	Human - randomized, double-blind,	35 hypertensive patients.<br>Three treatment periods of 6 weeks, separated by 2-week washout periods. The treatments will be	First trial investigating the potential anti-hypertensive benefit of dietary hemp protein plus bioactive peptide consumption in humans.

	augmentation index		crossover clinical trial. Methods paper.	consumed twice a day and consist of 25 g casein, hemp seed protein (HSP), or HSP plus HSP hydrolysate (HSP+)	*Results yet to be published.*
Hemp seed meal protein hydrolysate (HMH)	Blood pressure	Girgih et al 2014	Animal - rat	HMH was substituted for casein at 0.5 and 1.0% levels and fed to young growing rats for 8 weeks (preventive phase) or adult rats for 4 weeks (treatment phase)	Feeding of hypertensive rats with HMH resulted in attenuation of the normal increases in systolic blood pressure (SBP) with an average value of ~120 mmHg when compared to the casein-only group of rats (control) with a maximum of 158 mm Hg ($p < 0.05$).<br>Feeding adult rats (SBP ~145 mmHg) with same diets during a 4-week period led to significant ($p < 0.05$) reduction in SBP to ~119 mmHg in comparison with 150 mmHg for the control rats.<br>The results suggest that HMH with strong hypotensive effects in hypertensive rats could be used as a therapeutic agent for both the prevention and treatment of hypertension.
Hemp seed protein hydrolysate	Antihypertensive properties	Malomo et al 2015	In vitro	Hemp seed protein hydrolysates (HPHs) were tested for in vitro inhibitions of renin and angiotensin-converting enzyme (ACE), two of the enzymes that regulate human blood pressure.	The combination of the fast-acting HPH (1% alcalase) with the longer-lasting HPHs (2% and 4% pepsin) had highest effect on SBP.<br>The HPHs effectively reduced systolic blood pressure though they showed varying effectiveness.
Sesame seed protein (decorticated sesame seed extracted with isopropanol – DSS-Iso)	Growth performance, food efficiency ratio, plasma and tissue lipid,profile, plasma protein content, and erythrocyte membrane lipid profile	Sen et al 2001	Animal - rat	Rats fed 1) casein (control), 2) soybean meal, 3) decorticated sesame seed extracted with hexane (DSS-Hex) and compared with DSS-Iso	Rats fed a DSS-Iso-based diet showed body weight gain and food efficiency ratio similar to those of the control groups fed diets prepared with casein, soybean meal, and DSS-Hex. Rats fed a DSS-Iso-based diet showed significant decreases in plasma total cholesterol ($p < 0.01$), triglyceride ($p < 0.01$), and VLDL + LDL cholesterol ($p < 0.01$) concentrations in comparison to the rats fed diet containing casein.<br>DSS-Iso is a suitable edible protein like casein or soybean meal.
Sesame protein isolate	Lipids	Biswas A et al 2010	Animal – rat	18% sesame protein isolate with or without 2% cholesterol in comparison with casein fed to rats for 28 d.	Total cholesterol, LDL-cholesterol and triacylglycerol levels were significantly reduced in the sesame protein isolate and isolate containing cholesterol group than the corresponding control casein groups.<br>Sesame protein isolate decreases cholesterol concentration in plasma, increases HDL-cholesterol, and also decreases plasma and erythrocyte membrane lipid peroxidation with or without cholesterol fed diet in rats.
Sesame seed enzymatic protein hydrolysate	Antioxidant and antihypertensive markers	Aondona et al 2021	In vitro	Sesame protein hydrolysates and peptide fractions were assayed for in vitro antioxidant and antihypertensive properties compared to unhydrolyzed protein.	Sesame seed protein hydrolysates showed significant ($p < .05$) increases in the content of hydrophobic amino acids. Radical scavenging and metal ion chelation were also significantly ($p < .05$) enhanced by these treatments.

					Sesame seed peptides have demonstrated multifunctional potential to act as antioxidative and antihypertensive agents that could be utilized as ingredients for the development of novel functional foods and nutraceuticals.
Sunflower seed protein fraction extracted with isopropanol	Growth<br>Lipid profiles<br>Plasma protein content	Sen et al 2000	Animal – rat	Rats were fed sunflower seed protein fraction extracted with isopropanol (SSPF), sunflower meal (SM) or casein	Sunflower seed protein fraction resulted in a significant decrease in plasma cholesterol ($p < 0.05$) and LDL-cholesterol ($p < 0.02$) levels compared to the casein fed rats. Membrane phospholipid profile also showed a marked variation with the type of dietary protein. Rats fed SSPF and SM did not show much variation in plasma lipids, plasma proteins, liver and brain lipids and membrane phospholipid concentrations. Protein content, liver and brain lipid profile of the groups fed SSPF and casein were comparable, suggesting that the nutritional value of SSPF is better than SM and equivalent to that of casein.
Sunflower seed protein	Growth<br>Lipid profiles<br>Plasma protein content	Sen et al 2001	Animal – rat	Rats fed sunflower seed protein fraction or casein. Growth, plasma and tissue lipid profiles, plasma protein content, erythrocyte membrane lipid profile and organ weights of rats was measured.	The rats fed the sunflower seed protein fraction showed a significant decrease in plasma cholesterol ($p < 0.02$) and triglyceride ($P < 0.02$) concentrations compared to the casein-fed rats. The RE-fed rats also exhibited a significant difference in erythrocyte membrane phospholipids and phosphatidylcholine/phosphatidylethanolamine ratio. Rats fed the different dietary proteins did not show much variation in plasma protein content, liver and brain lipids and organ weights. These results demonstrate the hypolipidemic action of the sunflower seed protein fraction and that it can be considered a suitable edible protein like casein.
Walnut protein	Fatigue recovery. Blood urea nitrogen (BUN), blood lactic acid (BLA) and glycogen	Chen et al 2020	Animal - mice	Forty-eight ICR mice of specific pathogen free (SPF) were divided into a control group (group A) and low, medium and high walnut protein peptide content groups (group B, C and D). The mice swam until they were tired. Then the duration of exhaustive swimming, blood urea nitrogen (BUN), blood lactic acid (BLA) and glycogen of mice were measured.	Swimming time of group B, C and D was significantly higher compared to group A ($P < 0.05$), BLA was significantly lower than that of group A ($P <0.05$).<br>BUN was significantly lower than that of group A<br>content of glycogen was significantly higher compared to group A.<br>Walnut protein peptide can effectively inhibit the production of BLA and BUN, increase glycogen reserve, improve the endurance of mice, and promote fatigue recovery.
Walnut and peanut protein hydrolysates	Cognition properties incl memory	Li et al 2016	In vitro and animal - mice	The amino acid compositions and molecular weight distributions of soybean, walnuts and peanuts, the effects of various hydrolysates on H_2O_2-induced injury PC12 cells, and cognition of	The hydrolysates performed better than medication in memory ability and much stronger inhibitory activity against H_2O_2-induced toxicity.

				mice were investigated and compared against prescription medications.	Soybean, walnut, and peanut protein hydrolysates recommended as a potential food raw material for prevention or treatment of neurodegenerative disorders.
Walnut protein and walnut protein hydrolysate	Antioxidant and antihypertensive properties	Wang M et al 2016	In vitro and animal - spontaneously hypertensive rats	The physico-chemical properties, antioxidant and angiotensin-converting enzyme (ACE) inhibitory activities of WP, alcalase-generated walnut protein hydrolysate (AWPH) and trypsin-generated walnut protein hydrolysate (TWPH) were comparatively studied. Stability properties of the walnut protein hydrolysate (WPH) and the antihypertensive activity in spontaneously hypertensive rats (SHRs) were also investigated. No control group.	WPH significantly decreased the systolic blood pressure ($P < 0.05$).<br>Walnut protein hydrolysate superior properties to walnut protein.<br>The physico-chemical properties, antioxidant and angiotensin-converting enzyme (ACE) inhibitory activities of WP and alcalase-generated walnut protein hydrolysate were displayed in this study.
Walnut protein hydrolysates	Immune properties	Li J et al 2018	Animal - mice	Tested three types of walnut protein hydrolyzates-albumin, glutelin, and globin on the immune system of mice compared with control.	Those mice treated with different doses of walnut proteins showed improved immune indices, including organ index, spleen lymphocyte proliferation, macrophage activity, number of CD4+ and CD8+ T cells, immunoglobulin A (IgA) and secretory IgA content, and mRNA and protein expression levels of cytokine factors.<br>Potential as ingredient to boost the immune system.
Walnut protein hydrolysate	Renal function	Li Q Y et al 2018	Animal - hyperuricemic rats<br>In vitro	Tested the serum uric acid level and renal function in potassium oxonate-induced hyperuricemic rats in vivo as well as inhibit xanthine oxidase in vitro	Walnut meal hydrolysates and dephenolized walnut meal hydrolysates effectively decreased the serum uric acid level and protect the renal function in the rats.<br>The hydrolysates inhibited xanthine oxidase in vitro.
Walnut protein hydrolysate	Photoaging of the skin	Xu D F et al 2020	Animal – rats	The regulation of walnut protein hydrolysate (WPH) on inflammatory cytokine expression was examined in rat model.	WPH significantly reduced the expression level of inflammatory cytokines. The underlying mechanism of WPH ameliorating skin photoaging may be attributed to the synergistic modulation via reversing the inflammatory imbalance, suppressing the activation of the NF-kappa B signal pathway, stimulating procollagen type I synthesis, and inhibiting MMP-1 activities.
Peanut protein	Body composition and muscle contractile properties	Jacques et al 2010	Animal - rats	Thirty-two male rats were assigned to one of the following four diets:<br>1. casein<br>2. cod protein<br>3. peanut protein<br>4. casein + peanut protein (50:50) mixture<br>After 28d of ad libitum feeding and after 12-h fast, blood, liver and muscle were collected for	Rats fed with the low-quality protein, peanut protein, had lower body weight gain, body protein mass, soleus mass and liver weight than those fed with the high-quality dietary proteins, casein and cod protein.<br>Peanut protein increased plasma cholesterol and body fat mass compared with cod protein.<br>These results show that peanut protein intake alters body composition by reducing skeletal muscle mass and liver weight

				measurements of plasma and hepatic cholesterol and TAG, plasma glucose and insulin and contractile properties.	as well as muscle contractility and lipid metabolism. Adding a complete protein such as casein might partially counteract these adverse effects.
Hazelnut protein-derived peptide	Inflammatory markers	Ren et al 2018	In vitro	The anti-inflammatory activity of a novel peptide Leu-Asp-Ala-Pro-Gly-His-Arg (LDAPGHR) derived from Corylus heterophylla Fisch, in lipopolysaccharide (LPS)-stimulated macrophages was investigated.	Hazelnut protein-derived peptide exerted anti-inflammatory effects<br>Potential as a novel food ingredient.
Korean pine nut protein	Diabetes - glucose tolerance, liver indexes, glycolytic signalling	Liv et al 2019	Animal - type 2 diabetic mice	Testing water-soluble proteins of Korean pine nut (PNP) obtained from a dilute alkali extract on carbohydrate metabolism of type 2 diabetic mice on a model of diabetes induced using a high fat diet combined with streptozotocin.	Hypoglycemic effect of PNP at a middle dose was the most significant, which was 38.7% lower than that of control.<br>PNP significantly improved the oral glucose tolerance and liver indexes, increased the activity of the carbohydrate metabolism enzymes, and regulated the expression of the function of key genes for carbohydrate metabolism.<br>It had a positive effect on both insulin resistance and glycolytic/gluconeogenesis signalling.<br>Potential for PNP to be beneficial as a hypoglycaemic functional food in the treatment of type 2 diabetes mellitus.
Cashew nut protein hydrolysate	Antioxidant properties	Malomo et al 2020	In vitro	Antioxidant and renin-angiotensin system (RAS)-inhibitory protein hydrolysates derived from the enzymatic hydrolysis of cashew nut (CNP) and fluted pumpkin (FPP) proteins were investigated using in vitro techniques.	Renin inhibition was significantly ($p<0.05$) increased by ultrafiltration from 45.7 and 62.1% to similar to 82.4 and 96.5% for fluted-pumpkin protein hydrolysates and cashew nut protein hydrolysate, respectively.<br>The strong antioxidant properties coupled with RAS inhibition make CNP hydrolysates and their low molecular weight peptides potential ingredients to formulate health-promoting foods.

Supplementary Table S7. Legume (non soy) Protein, Human Studies

Protein type & form	Biomarker / Health measures	Reference	Study type	Study summary	Outcome
Fava bean and peas	Appetite sensation<br><br>Ad libitum energy intake	Nielsen et al 2018	Human<br>Men, Healthy stable weight (BMI 18.5-30.0 Kg/m2), Age: 18-50<br>N=35<br>Single blinded, randomised acute, cross over	Three iso-caloric, macro-nutrient balanced, fibre-matched test meals based on:<br>1) Fava beans/split peas,<br>2) Pork & veal with pea fibre, and<br>3) Egg with pea fibre and<br>4) Control - iso-caloric egg-based meal without fibre matching.<br>Subjective appetite sensation assessment using visual analogue scale at baseline and every 30 min for 3 hours until an ad libitum lunch.<br>A standard dinner the night before testing<br>A weighed food record from completion of the test until midnight to evaluate compensatory energy intake.	No significant difference ($p>0.05$) in ad libitum energy intake<br>No significant difference ($p>0.05$) in subjective appetite sensation composite score calculated from satiety, hunger, fullness, and prospective food consumption (PFC) and individual scores for satiety, hunger, fullness, and desire to eat meat or fish, salty, sweet or fatty food between the four meals. This did not change when the data was analysed as iAUC or iAOC.<br>The PFC rating was significantly higher for the fava bean/split peas meal compared to the egg fibre meal (16 ± 2.2 mm vs. 11.4 ± 2.3 mm, $p < 0.05$).
Fava bean and peas	Subjective appetite sensation<br>Satiety	Kristensen et al 2016	Human<br>N=43<br>Healthy young men, normal weight<br>Randomized, double blind, placebo controlled, three way cross over	Two macro-nutrient and calorie matched test meals;<br>1) High protein (HP) meat based on pork and veal<br>2) High protein (HP) legume based on fava beans and split peas<br>A third energy and fat-matched<br>3) Low protein (LP) legume meal based on fava beans and split peas<br>Different fibre content (6 g, 23 g and 10 g fibre for HP-meat, HP-legume and LP-legume meals respectively)<br>Subjective appetite sensation assessment using visual analogue scale (VAS) at baseline and every 30 min until an ad libitum lunch after 3 hrs	HP-legume induced significantly lower ($p<0.05$) composite appetite score, hunger, prospective food consumption and significantly higher ($p<0.05$) fullness compared to HP-meat and LP-legumes.<br>HP-legume induced significantly lower ($p<0.05$) composite appetite score, hunger, prospective food consumption and significantly higher ($p<0.05$) fullness compared to HP-meat after compensating for palatability.<br>A significantly higher ($p<0.05$) satiety for HP-legume compared to HP-meat<br>A 12% and 13% lower ad libitum energy intake observed for HP-legumes compared to HP-meat and LP-legumes respectively($p<0.05$)
Pea, lentil, rice and maize protein isolates	Heme iron absorption	Weinborn et al 2015	Human, 30 adult females, Age:35-45, two studies, N=15 per study	Heme Fe labelled with radioactive Fe isotopes was prepared from rabbit and calf blood, which were injected earlier with radioactive Fe isotopes (55Fe, 59Fe)<br>Study 1: Participants ingested 55Fe labelled heme iron alone on the first day as control, followed by 59Fe labelled heme with zein suspension on the second day, 55Fe labelled heme iron with gliadin suspension on the 14th day and 59Fe labelled heme with glutelin suspension on the 15th day. Blood samples were taken	The average Heme Fe absorption in study 1 was 6.2%, which slightly increased with co-ingestion with zein and gliadin and slightly decreased with glutelin, however none of them were significant ($p>0.05$)<br>The average heme Fe absorption alone in study 2 was 11%. Pea and lentil protein isolates did not have significant effect on absorption whereas soy protein concentrate significantly decreased heme Fe absorption ($p<0.02$)

				from the subjects on the 14th and 28th days to determine Fe absorption.<br>Study 2: Participants ingested 55Fe labelled heme iron alone on the first day as control, followed by 59Fe labelled heme with soy protein concentrate suspension on the second day, 55Fe labelled heme iron with pea protein isolate suspension on the 14th day and 59Fe labelled heme with lentil protein isolate suspension on the 15th day. Blood samples were taken from the subjects on the 14th and 28th days to determine Fe absorption.	
Lentils, chickpeas, peas and beans (whole)	Inflammation biomarkers: high sensitivity C-reactive protein (HS-CRP), IL-6 and TNF-α	Hassein pour-Niazi et al 2015	Human, N=31, type 2 diabetic, 24 women, 7 men; average age=50 to 75, average BMI=25 to 30 kg/m2, randomised, crossover trial	Participants randomly assigned to one of the following two intervention diets for 8 weeks<br>Legume free therapeutic lifestyle change (TLC) diet.<br>Isoenergetic non-soy legume-based TLC diet, macronutrient and fibre matched except that two servings of meat are replaced by lentils, chickpeas, peas or beans 3d per week. Half cups of legumes replaced one serving of meat.<br>After a wash out period of 4 weeks, the groups switched to the alternate diet for another 8 weeks.<br>Fasting blood samples taken at baseline and after the 8 weeks intervention periods to analyse concentration of inflammation markers.<br>There was over 90% of compliance to the intervention diets based on the dietary records of the participants.	Both TLC diets significantly reduced the concentration of inflammation biomarkers (High sensitivity C-reactive protein, IL-6 and TNF-α). The non-soy legume TLC diet resulted in a significantly lower ($p<0.05$) plasma concentration of inflammation biomarkers compared to the non-legume diet TLC diet (HS-CRP decreased by 1.3 and 1.7 mg/L respectively from baseline values of 3.9 and 4.0, p=0.019, IL-6 decrease by 1.2 and 1.6 pg/L respectively from baseline value of 14.8, p=0.018, TNF-α decreased by 1.3 and 1.8 pg/L respectively from a baseline value of 8.1, p=0.018)
Lupin protein hydrolysate (LPH)	Immune and antioxidant responses of human peripheral blood mononuclear cells (PBMCs): pro-inflammatory Th1 [interleukin (IL)-2, interferon-γ (IFN-γ) and tumor necrosis factor (TNF)] and anti-inflammatory Th2 (IL-4 and IL-10) cytokines, antioxidant capacity	Cruz-Chamorro et al 2021	Human, N=33, Age= 18-50	A beverage (LPHb) containing lupin protein hydrolysate was prepared in an emulsion format<br>Participants consumed 200 mL of the beverage containing 1 g of LPH in the morning before any other food for 28 days<br>Fasting peripheral blood and urine samples analysed before, during (14 days) and after LPHb ingestion.	No significant effect of LPHb ($p>0.05$) on total cholesterol, LDL, HDL and total glycerides.<br>A slight (4.2%) but significant reduction ($p\leq0.05$) in LDL/HDL ratio of the population due to the significant reduction in LDL/HDL ratio of the male participants (15%).<br>A significant reduction in TC and LDL in males with higher baseline risk factors for CVD (high BMI, high TC, high Caselli risk index I (TC/LDL) and II (LDL/HDL).<br>A significant reduction in phytohemagglutinin-P (PHA) stimulated production of Th1 pro-inflammatory cytokines IL-2 (~40%), IFN-γ (25%) and TNF(~45%).<br>A significant increase in TAC (~12%) and ORAC (~7%) antioxidant capacities of PBMCs were also observed.

	Total cholesterol TC), LDL/HDL ratio				
Lupin protein concentrate	Plasma lipids: total cholesterol, LDL and HDL cholesterol, non-HDL cholesterol<br>Apolipoprotein A-I<br>Apolipoprotein B<br>C-reactive protein<br>Fasting blood glucose<br>Insulin<br>Blood pressure	Pavanello et al 2017	Human, moderately dyslipidaemic, N=50, Age=45-75, men and postmenopausal women, BMI= 25-32 kg/m2 randomised parallel, double blind, single centre	Half of participants were fed a normal caloric and low lipid diet together with 30 g/day lupin protein concentrate for 12 weeks. The other half fed 30 g lactose-free skimmed milk powder per day together with the same low lipid diet as above.<br>Major nutrient intake in the two diets were similar except that different diets were designed for female and male participants.<br>Clinical assessments were conducted at screening, baseline and every four weeks until the end of the study.	Total cholesterol level significantly decreased to the same degree in both the milk and the lupin diets (6.7% & 7.2% respectively). However, LDL decrease was only significant in the lupin diet (8% reduction) and so was the reduction in non-HDL cholesterol (8% reduction) and proprotein convertase subtilisin/kexin type 9 (PCSK9) (12.7% reduction).
Lupin protein isolate	Total cholesterol<br>LDL cholesterol<br>HDL cholesterol<br>Oxidised LDL cholesterol<br>High sensitivity C-reactive protein (hs-CRP)<br>Triacylglycerols<br>Urea<br>Uric acid<br>homocysteine	Bahr et al 2015	Human, N=72, Age=18-80, hypercholesterolemic subjects (total cholesterol ≥5.2 mmol/L), randomized, controlled, double-blind, three-phase crossover study	Three intervention study periods each 28 days separated by 6 weeks washout periods<br>Three intervention diets:<br>1) Lupin protein (LP) intervention: 25 g/d lupin protein isolate (81.6% protein) containing 2.5 g arginine incorporated into complex food products<br>2) Milk protein (MP) intervention: 25 g/d milk protein isolate (75% casein, 25% whey protein) containing 0.9 g arginine incorporated in complex food products<br>3) Milk protein intervention plus arginine (MPA) intervention: 25 g/d milk protein isolate plus arginine (1.6 g capsule) in complex products to compensate for the higher arginine level in lupin<br>Mannitol placebo capsules in LP and MP interventions.<br>Products: Bread, rolls, scalded sausage, vegetarian spread<br>The intervention products had similar levels of proteins, carbohydrates, fats & fibre.<br>Fasting blood samples analysed prior to and after 28d interventions.	Total cholesterol significantly decreased after LP and MPA diet ($p<0.05$) by 4.3% and 5.3% respectively. No significant decrease after MP diet<br>LDL cholesterol significantly ($p<0.05$) decreased after the LP and MPA diets by 3.6% and 3.8% respectively, HDL significantly decreased by 3.6% and 6.9% respectively whereas triacylglycerols decreased only after LP. The result indicates that the effect of LP on blood lipids could be due to its higher arginine level.<br>A significant increase in oxidised LDL after LP diet.<br>The relative change in total cholesterol after LP intervention was significantly higher for subjects with severe hypercholesterolemia (>6.6 mmol/L) compared to those with moderate hypercholesterolemia (5.5 to 6.6 mmol/L). The decrease in total cholesterol level was around 6% in the severe compared to ~2% in the moderate group and the change in the LDL was around 0.3% in the moderate compared to ~5.8% in the severe.<br>Urea increased after all interventions and the increase was significantly higher in the MPA intervention.<br>Homocysteine was significantly lower after LP intervention compared to MP and MPA. A significant increase in homocysteine observed after MP.<br>Uric acid significantly decreased after LP intervention.

Lupin protein isolate (91.7% protein, low fat), and pea protein isolate (90.8% protein, low fat, low fibre)	Total cholesterol<br>LDL cholesterol<br>HDL cholesterol	Sirtori et al 2012	Human, men and postmenopausal women, moderately hypercholesterolemic (Total cholesterol >2200mg/L, TAG < 2000mg/L), N=175, a randomised double blind, parallel group design	All participants had a hypolipidemic diet for 4 weeks<br>Baseline assessment after the 4wks period and then after the four weeks intervention diet. Overnight fasting blood samples<br>Participants randomly grouped into 7 dietary interventions, 25 in each group<br>Each group consumed two bars containing a specific protein-fibre combination for 4 weeks<br>1) casein + cellulose: control<br>2) lupin protein+ cellulose<br>3) pea protein+ cellulose<br>4) casein + oat fibre<br>5) casein + apple pectin<br>6) pea protein+ oat fibre<br>7) pea protein+ apple pectin<br>Slightly higher protein and moisture content in the casein bars. Matching fibre, carbohydrate and fat contents	Significant reductions in total cholesterol were observed in the case of casein & apple pectin (5·3 %), lupin protein & cellulose (4·2 %), pea protein & oat fibre (4·7 %) and pea protein & apple pectin (6·4 %) interventions, whereas no significant change was observed with the casein & cellulose interventions.<br>A significant reduction in LDL-C observed in the pea protein-apple pectin and the pea protein-oat fibre interventions.<br>Minimal changes in HDL and TAG<br>The result indicates the hypocholesteremia effect of lupin protein whereas pea protein appears to have effect in combination with soluble fibres.
Lupin γ-conglutin	Blood glucose<br>Insulin	Bertoglio et al 2011	Human: healthy adults, N=15, placebo controlled	Subjects given single doses of three different test products containing 157.5, 315 and 630 mg γ-conglutin, respectively, and a placebo, with no γ-conglutin added 30 min before a carbohydrate meal (85 g rice containing 75 g carb).<br>Study over 7 weeks.<br>Blood samples drawn 10 min before ingestion of the test product, after ingestion of the test product, after 15 min, 30 min (before carb intake), every 30min until 2hr and after 3hrs	A statistically significant reduction in blood sugar and similar degree of reduction after 60 min compared to placebo with all doses, a significant reduction by the two higher doses at 90 min and a significant reduction maintained by the highest dose at 120 min.<br>An increase in insulin secretion at all doses at 60 min followed by decay of similar pattern at all doses.<br>The blood glucose level as AUC showed a statistically significant reduction of 25% and 21% at the intermediate and the highest γ-conglutin dose respectively.
Lupin protein isolate	Cholesterol in plasma and lipoproteins	Weisse et al 2010	Human, hypercholesteremic, N=43, randomised, double blind, placebo controlled, parallel study	10 day run-in period and 6 weeks study period<br>Participants received either of the following snacks per day in two servings:<br>1) Control: 35g casein incorporated into a snack bar<br>2) Test: 35g isoflavone free lupin protein isolate containing 16 mg alkaloids per kg protein isolate<br>Participants advised to consume the bars as a replacement of an isocaloric snack in their diet<br>Blood samples taken at screening, at baseline and at the end of the intervention	A significant decrease in total cholesterol in both interventions (8.1% and 8.8% respectively with casein and lupin) with no difference in the level of reduction.<br>A significant reduction in LDL in the lupin group (8.6%) whereas no change was observed in the casein group.<br>A significant reduction in HDL in the casein group (10%), while no change was observed in the lupin group.<br>A significantly lower LDL/HDL observed in the lupin intervention compared to casein at the end of the 6 wks.
Pea protein hydrolysate	HbA1c<br>Fasting glucose<br>Postprandial glucose and insulin	Chauhan et al 2021	In vitro, mice, & human: pilot double blinded, placebo controlled, healthy adults	A pea protein hydrolysate designed using an Artificial intelligence model and prepared via enzymatic hydrolysis of pea protein powder<br>Subjects randomly assigned to placebo (Avicel crystalline cellulose) (N=20), protein hydrolysate control (Rice peptide	A small but significant decrease of 0.12% observed in HbA1c in the NRT_N0G5IJ intervention compared to baseline. No such effect was observed in the placebo or rice hydrolysate interventions.

			with elevated HbA1c (5.7 to 6.4%), Age: 18-75, BMI: 20-35 kg/m2, N=63	network (NPN)) (N=20) and pea protein hydrolysate (NRT_N0G5IJ) (N=23) interventions.<br>15g of the test product consumed after dissolving in 200 mL water per day for 12 weeks.<br>Fasting blood samples taken at baseline, wk 4, wk 8 and wk 12.	
Pea seed albumin	Inflammatory markers - cytokines, inducible enzymes, metalloproteinases, adhesion molecules, and toll-like receptors, as well as proteins involved in maintaining the epithelial barrier function	Utrilla et al 2015	Animal – mice	5 groups: one noncolitic and four with induced colitis.<br>1. No colitis – no treatment<br>2. Colitis – no treatment<br>3. Colitis – treated with pea seed extract<br>4. Colitis reated with albumin fraction from pea seed extract<br>5. Colitis – treated with soy	Colitis was ameliorated in the pea seed extract treated group and the albumin fraction from pea seed extract treated group.<br><br>These 2 groups plus the soy treated group had their bacterial counts restored to values in healthy mice.
Rice, pea and oat proteins	Glycaemic response: postprandial glucose, insulin, triglycerides, glucose-dependent insulinotropic peptide (GIP), glycogen-like peptide-1 (GLP-1) and appetite	Tan et al 2018	Human, N=20, Age=21-45, BMI:18.5-24.9kg/m2, healthy Chinese men, randomised crossover, acute feeding trial	Four test products:<br>1) Chocolate beverage alone (50 g carb): control<br>2) Chocolate bev+25 g oat protein<br>3) Chocolate bev+25 pea protein<br>4) Chocolate bev+25 g rice protein<br>Test products had matching macronutrient profile except the lower calorie and protein content of the chocolate beverage.<br>Subjects ingested the test beverage after overnight fast.<br>A washout period of at least 7 days between test beverages.<br>Subjective appetite ratings and blood samples collected before ingestion and at fixed intervals for 180 min	A significantly higher insulin response (as iAUC) in the pea and oat protein compared to control<br>No significant effect of the test proteins on postprandial glucose.<br>No significant effects of the proteins on other tested parameters including appetite.
Pea, oat and potato protein blend	Satiety<br>Appetite<br>Energy intake	Dougkas et al 2018	Human, randomised single blind, crossover, N=28, healthy male, BMI: 20-29.9 kg/m2	Three isoenergetic, macronutrient matched rice pudding containing<br>1) Milk protein (AP)<br>2) A mixture of plant proteins (peas, oat, potato) (VP)<br>3) A 50:50 mixture of animal and plant proteins (MP)<br>4) A carb rich meal: control<br>Participants given the test meal at breakfast an appetite ratings (using visual analogue scale (VAS)) and blood samples taken every 30 and 60 min respectively until the ad libitum lunch after 3.5 hrs.	A significantly lower subjective desire to eat in the MP diet compared to control.<br>A significantly higher fullness in the VP and MP diet compared to control.<br>A significantly lower appetite in the MP diet compared to control.<br>No significant difference between AP, VP and MP in all the parameters.<br>No significant difference in ad libitum energy intake between the four meals.

				Energy intake and appetite recorded hourly afterwards for the remainder of the day.	Glucose and insulin responses significantly higher in the control meal compared to the protein breakfasts.
Four protein hydrolysates from vegetable (pea, gluten, rice and soy) and two protein hydrolysates from animal origin (whey and egg)	Postprandial plasma glucose, glucagon, insulin and amino acids	Claessens et al 2009	Repeated-measures design with Latin square randomization and single-blind trials	Postprandial plasma glucose, glucagon, insulin and amino acids were determined over 2 h. RESULTS: All protein hydrolysates induced an enhanced insulin secretion compared to maltodextrin alone and a correspondingly low plasma glucose response. A significant difference was observed in area under the curve (AUC) for plasma glucagon between protein hydrolysates and the maltodextrin control drink (P<0.05).	High amino-acid-induced glucagon response does not necessarily go together with low insulin response. Protein hydrolysate source affects AUC for glucagon more profoundly than for insulin.
Pea protein isolate	Blood glucose<br>Appetite<br>Ad libitum Food intake	Mollard et al 2014	Human, healthy young men, N=15, Age=18-35, single blind randomised crossover trial	Treatments:<br>1) Pea hull fibre (94% fibre), 7g fibre<br>2) Pea protein (82% protein), 10 g protein<br>3) Pea hull fibre (7 g) + pea protein (10 g)<br>4) Canned yellow peas containing 7 g fibre and 10 g protein<br>5) Noodle and tomato sauce: control<br>The pea hull and the pea protein were consumed with noodle and tomato sauce and the yellow peas with tomato sauce<br>Treatments matched for energy but higher available carbohydrate in control and fibre meals.<br>The participants consumed the test meals in a random order 1 wk apart after an overnight fast<br>Baseline blood glucose (BG) and subjective appetite using visual analogue scale (VAS) measured before ingestion of the test meal and 15, 30, 45, 75 and 135 min after the meal.<br>At 135 min, the participants were fed ad libitum pizza lunch.	No significant effect of treatment on ad libitum food intake.<br>There was no significant effect of treatment on pre-pizza lunch subjective appetite (AUC).<br>Treatment had a significant effect on pre-pizza and cumulative BG (AUC). Treatment had no effect on post-pizza BG.<br>Among the treatments, only protein + fibre and yellow peas had significant effects on BG.<br>Time and treatment and treatment x time interactions were also significant for both appetite and BG
Yellow pea protein isolate	Blood glucose (BG)<br>Appetite<br>Ad libitum Food intake (FI)	Smith et al 2012<br><br>*Similar group as above*	Human, healthy young men, Age: 20-30 years, N=19 Expt 1, N=20 Expt 2, single blinded, randomised, repeated measure design	Treatments:<br>1) Pea protein isolate (10g): P10<br>2) Pea protein isolate (20g): P20<br>3) Pea hull fibre (10g): F10<br>4) Pea hull fibre (20g): F20<br>5) Control: tomato soup<br>Treatments given with tomato soup, volume, Na, fat matched. Similar levels of available carbohydrate.<br>Subjects given one treatment meal per week, one week apart.<br>Subjects fasted overnight and were given a standardised high carbohydrate breakfast and were given the treatment meal after	In expt 1, a significantly lower FI compared to all other treatments in P20 (20 g pea protein).<br>The mean BG pre-pizza meal was significantly lower in P10 and P20 treatments compared to control.<br>The mean BG post pizza meal was significantly lower in P20 compared to control and F10 in Expt 1.<br>No treatment effect on the mean BG observed in Expt 2 pre pizza meal.<br>Treatment effect on BG post pizza meal but no significant difference between treatments.<br>Treatment did not have significant effect on subjective appetite in both experiments pre and post pizza meal.

				2hr. Baseline assessment of appetite (VAS) and BG at time zero before ingesting the test meal.<br>Expt 1: Assessment of FI at pizza meal served 30 min after the test meal, BG and appetite assessment at 15 and 30 min and periodically between 50 min and 170 min post pizza meal<br>Expt 2: Assessment of FI at pizza meal served 120 min after the test meal, BG and appetite assessment periodically between 15 min and 120 min pre-meal and post pizza meal up to 200 min.	
Pea protein hydrolysate (PPH) (< 3 KDa peptides)	Blood pressure (BP)	Li et al 2011	Rats and humans, hypertensive (systolic BP 125-170 mmHg), N=7, Age:30-55, randomised, double blind, placebo controlled, crossover	Three week trial:<br>1) Placebo: 50 mL orange juice<br>2) PPH1:1.5 g PPH per day in three 0.5 doses in 50mL orange juice at breakfast, lunch and dinner<br>3) PPH2: 3 g PPH per day in three does 1g each in 50 mL orange juice<br>2 week washout period<br>BP taken three times a day at the same time	PPH2 resulted in a significant reduction of systolic BP of 5 and 6 mmHg after wk2 and wk3 of the study.<br>The rat study indicated the observation is due to effect on renal renin mRNA levels which may be responsible for the observed decreases in plasma angiotensin II concentrations.
Pea protein2	Appetite<br>Food intake (FI)<br>Glucagon like peptide 1 (GLP-1)<br>Cholecystokinin (CCK)<br>Peptide YY (PYY)	Geraedts et al 2011	Human, N=10 obese (BMI:33.4±1.4, Age:41± 6) and N=10 lean (BMI: 23±0.7, Age: 25±2), male	Treatments:<br>1) Pea protein solution: 250 mg/kg as 0.4 mL/kg body weight<br>2) Placebo: 0.4 ml/kg body weight similarly flavoured as the test drink<br>Administered orally (OPA) or intraduodenally (IPA)<br>Four visits 1 week apart, standardised dinner prior to visit and overnight fast.<br>Fasting blood sample taken prior to administration of the treatment.<br>Subjective appetite and other biomarkers assessed periodically over 2hrs followed by an ad libitum meal (lasagne Bolognese).	Significant higher CCK levels 10 and 20 min after IPA and also compared to OPA in obese subjects.<br>GLP-1 levels increased after 90 and 120 min following IPA compared to OPA in obese subjects.<br>A significant decrease in ad libitum FI decreased in both lean and obese subjects after IPA.<br>In obese subjects, FI was significantly lower in IPA compared to OPA.<br>Delivery of intact pea protein reduces food intake and that seems to be more effective in obese subjects.
					•
Pea protein	Short term satiety:<br>Combined satiety score (CSS) calculated from VAS assessment<br>Blood glucose	Abou-Samra et al 2011	Human, two expts, N=32 in both, male, Age: 20-35, BMI: 20-27 kg/m2, randomised, single blind, crossover	**Expt1:** 7 study sessions (1 training, 6 trial), sessions 2 days apart, subjects advised to eat similar meals the evening before trial session<br>Test products in beverage form:<br>1) Pea protein: 20 g in 200 mL water<br>2) Casein: 20 g in 200 mL water<br>3) Whey: 20 g in 200 mL water<br>4) Egg albumin: 20 g in 200 mL water<br>5) Maltodextrin: 20 g in 200 mL water	**Expt 1:**<br>Energy intake after ad libitum meal significantly lower in the case of casein and pea protein preload compared to other test meals.<br>CSS significantly higher in casein and pea protein preload compared to others. Lowest CSS with water preload and significantly higher than water but similar for whey, egg albumin and maltodextrin preloads.

				6) +50 mL washout water<br>7) Water: control (250 mL)<br>The test meal was followed by an ad libitum meal (Crème Budwig) 30 min later in Expt 1.<br>Subjective appetite assessment (VAS) before and after the test meal every 10 min until the ad libitum meal and twice afterwards.<br>Blood glucose measurements prior to test meal, periodically until and after the ad libitum meal for 2 hrs.<br>**Expt2:** 5 sessions: 1 training, 4 test, all test foods in expt1 except egg protein and maltodextrin, isocaloric<br>Ad libitum breakfast: Bircher Muesli served right after the test meal.<br>VAS assessment at baseline and for 45 min after the ad libitum meal.	A significantly lower BG after whey preload compared to all the other preloads.<br>**Expt 2:**<br>No significant difference in ad libitum energy intake.<br>No significant difference in CSS between the four preloads.<br>The impact of protein preload (pea and casein) significant if there is a time difference between preload (starter meal) and ad libitum meal.
Pea protein hydrolysate	Satiety<br>Satiety hormones: Glucagon like peptide 1 (GLP-1)<br>Cholecystokinin (CCK)<br>Peptide YY (PYY)<br>Blood glucose	Diepvens et al 2008	Human, randomised crossover trial, with two expts, N=39, healthy adults, BMI:25-31 kg/m2, Age:18-60	Test products- in a shake format<br>1. Whey protein (WP):15g<br>2. Pea protein hydrolysate (PPH):15g<br>3. WP+PPH: 7.5g+7.5g<br>4. Milk protein (MP, 80% casein, 20% WP): 15g<br>Isocaloric and macronutrient matched test products.<br>**Expt 1:** Assessment of appetite, satiety related hormones and glucose periodically over 4hrs following ingestion of test meals<br>**Expt 2:** Assessment of appetite periodically over 7 hrs and energy intake (EI) during an ad libitum lunch (Egg salad sandwich with Turkish bread) served 180 min after ingestion of test meal<br>Four test sessions, 1 wk between sessions, subjects arrived after overnight fasting	**Expt 1:**<br>A significantly lower hunger score for PPH shake compared to WP at 240 min and greater satiety and fullness after 180min compared to MP.<br>No significant effect on insulin, significantly higher CCK and GLP-1 in MP compared to the other interventions.<br>**Expt 2:**<br>No significant difference was observed in food intake after 180 min between the treatments and no significant difference in hunger scores.<br>PPH resulted in a greater satiety, and fullness, and a smaller desire to eat and thirst, at various timepoints (30, 60 or 180 min) compared to either MP or WP + PPH ($p<0.05$).<br>WP resulted in a greater satiety, and fullness at 30 and 60 min and 60 min after lunch compared to MP or WP + PPH ($p<0.05$).<br>No strong correlation observed between subjective satiety and satiety hormones.
Lentil protein and other fractions	Satiety<br>Blood glucose (BG)<br>Insulin	Fabek et al 2016	Human, randomised, crossover, repeated measure trial, two expts, Healthy men, N=48	Test products (300 mL each)<br>1. Tomato soup- control<br>2. Tomato soup+20 g lentil protein isolate (75% protein)<br>3. Tomato soup+20 g lentil protein concentrate (55% protein)<br>4. Tomato soup+20 g lentil starch (60% starch)<br>5. Tomato soup+20 g lentil fibre (55% fibre)	**Expt 1:**<br>The lentil starch test product significantly ($p<0.0001$) increased pre-pizza meal BG.<br>The lentil protein isolate and concentrate significantly ($p<0.0001$) decreased post-pizza meal BG without increasing insulin.

	**Expt 1:** ad libitum pizza meal after 30 min<br>**Expt 2:** ad libitum pizza meal after 120 min	None of the treatments had a significant effect on pre-meal satiety measures. However, post-pizza meal subjective appetite was significantly lower in the case of lentil protein isolates and concentrates ($p<0.05$).<br>**Expt 2:**<br>The lentil starch treatment resulted in a significantly lower subjective appetite pre and post-pizza meal ($p<0.05$) but significantly increased BG.<br>Lentil protein preparations resulted in a higher reduction in BG after 120 min compared to 30 min.
